# Putative bovine topological association domains and CTCF binding motifs can reduce the search space for causative regulatory variants of complex traits

**DOI:** 10.1186/s12864-018-4800-0

**Published:** 2018-05-24

**Authors:** Min Wang, Timothy P. Hancock, Amanda J. Chamberlain, Christy J. Vander Jagt, Jennie E. Pryce, Benjamin G. Cocks, Mike E. Goddard, Benjamin J. Hayes

**Affiliations:** 1 AgriBio, Centre for AgriBioscience, Agriculture Victoria, Melbourne, VIC Australia; 2 School of Applied Systems Biology, La Trobe University, Melbourne, VIC Australia; 3 DataGene Ltd, Bundoora, VIC 3083 Australia; 4 Faculty of Veterinary and Agricultural Sciences, University of Melbourne, Parkville, Melbourne, VIC Australia; 5 Queensland Alliance for Agriculture and Food Innovation, University of Queensland, St Lucia, QLD Australia

**Keywords:** Topological association domains, CTCF binding motifs, Allelic-specific expression, Allele-specific expression quantitative trail loci, Expression quantitative trail loci, Functional annotation, Cattle

## Abstract

**Background:**

Topological association domains (TADs) are chromosomal domains characterised by frequent internal DNA-DNA interactions. The transcription factor CTCF binds to conserved DNA sequence patterns called CTCF binding motifs to either prohibit or facilitate chromosomal interactions. TADs and CTCF binding motifs control gene expression, but they are not yet well defined in the bovine genome. In this paper, we sought to improve the annotation of bovine TADs and CTCF binding motifs, and assess whether the new annotation can reduce the search space for *cis-*regulatory variants.

**Results:**

We used genomic synteny to map TADs and CTCF binding motifs from humans, mice, dogs and macaques to the bovine genome. We found that our mapped TADs exhibited the same hallmark properties of those sourced from experimental data, such as housekeeping genes, transfer RNA genes, CTCF binding motifs, short interspersed elements, H3K4me3 and H3K27ac. We showed that runs of genes with the same pattern of allele-specific expression (ASE) (either favouring paternal or maternal allele) were often located in the same TAD or between the same conserved CTCF binding motifs. Analyses of variance showed that when averaged across all bovine tissues tested, TADs explained 14% of ASE variation (standard deviation, SD: 0.056), while CTCF explained 27% (SD: 0.078). Furthermore, we showed that the quantitative trait loci (QTLs) associated with gene expression variation (eQTLs) or ASE variation (aseQTLs), which were identified from mRNA transcripts from 141 lactating cows’ white blood and milk cells, were highly enriched at putative bovine CTCF binding motifs. The linearly-furthermost, and most-significant aseQTL and eQTL for each genic target were located within the same TAD as the gene more often than expected (Chi-Squared test *P*-value < 0.001).

**Conclusions:**

Our results suggest that genomic synteny can be used to functionally annotate conserved transcriptional components, and provides a tool to reduce the search space for causative regulatory variants in the bovine genome.

**Electronic supplementary material:**

The online version of this article (10.1186/s12864-018-4800-0) contains supplementary material, which is available to authorized users.

## Background

Identifying causal mutations is essential for improving the accuracy and reliability of genomic selection [1]. This identification task is challenging because the large scale linkage disequilibrium and small effects of most mutations in the bovine genome can drive a false discovery in a genome-wide association study (GWAS) [2]. To distinguish the causal mutations from noise, gene expression can be utilised to identify genomic loci that shape the trait of interest. An expression quantitative trait locus (eQTL) is a heterozygous locus that is associated with total changes in a gene’s expression in a group of individuals. An allele-specific expression quantitative trait locus (aseQTL) is a heterozygous locus that explains allele-specific expression of a particular gene transcript in a group of individuals. Here, the heterozygous locus is called eSNP or eVariant, and the gene that displays expression variation is called eGene [3–7]. Both aseQTL and eQTL mappings require high computational capacities to test association between any eSNP and any eGene genome-wide. To reduce the computing time and space, we propose to take into account the transcriptional regulatory structure that confines the scope of chromosomal interactions, so we may only need to test *cis*-association between eSNPs and eGenes under the same transcriptional control.

Topological association domain (TAD) is a type of regulatory structure that has never been described in the bovine genome. TADs are empirically defined from data that are produced from Hi-C or other chromatin conformation capture technologies [8–13]. TADs partition the genome by contact frequencies, where chromosomal regions within the same domain self-interact much more frequently than between domains. TADs are highly conserved between cell types and species [14–16], and the disruptions of TADs were found to cause disease-related gene expression by exposing genes to inappropriate regulatory elements [13, 17, 18]. A TAD boundary is defined as genomic interval, no larger than 400 Kb, between adjacent TADs where self-interactions decrease [14]. The TAD boundaries were found to be enriched for housekeeping genes, transfer RNA (tRNA) genes, tri-methylation of lysine 4 on histone H3 (H3K4me3), short interspersed nuclear elements (SINEs), DNase hypersensitive sites, and CTCF binding sequences [9, 14, 19, 20].

The CCCTC-binding factor (CTCF) is an 11 zinc-finger protein that mediates transcriptional regulation. CTCF can bind to evolutionarily conserved DNA sequences to prevent inappropriate enhancer-promoter interactions, and these conserved CTCF binding sequences were found to be enriched at TAD boundaries [14, 21]. CTCF can also bind to evolutionarily non-conserved DNA sequences to facilitate unique enhancer-promoter interactions at various steps of the transcriptional process, and these diverse CTCF binding sequences were often distributed within TADs [14, 22, 23]. The CTCF protein has a highly conserved amino acid sequence that is 93% identical from avian to human, and its functional diversities are achieved through different combinations of zinc finger domains binding to DNA sequences [24]. These DNA sequences have distinctive patterns, which are called CTCF binding motifs. Some CTCF binding motifs were found to be highly conserved across 180 million years of evolution [25]. Similar to TADs, mutations at CTCF binding motifs are commonly deleterious [26].

In this study, we aimed to assist in reducing the search space for causative regulatory variants by identifying TADs and CTCF binding motifs in the bovine genome. We mapped bovine TADs and CTCF binding motifs based on homology with humans, mice, dogs and macaques. We validated mapped bovine TADs by known biological hallmarks, and validated mapped bovine CTCF binding motifs by public databases for transcription factor (TF) binding motifs. We showed that mRNA expression profile variation across multiple lactating cows’ tissues and cells were confined by TADs and CTCF binding motifs. We suggest that our putative TADs and CTCF binding motifs could reduce the search space for causative regulatory variants in the bovine genome.

## Results

### Mapping mammalian topological association domains to the bovine genome

To create a library of putative bovine topological association domains (TADs), we directly mapped mammalian TADs to the bovine genome, and tracked the changes of mammalian TADs in each step of the mapping. When genomic conversion was to an updated version of reference assembly of the same species (hg18 to hg19), query TADs recovered well in the target genome (Additional file 1: Table S1). Over 89% of query TADs mapped uniquely to a single location in the target reference assembly without splitting within (intra-chromosomal) or across (inter-chromosomal) chromosomes. The query and target TADs not only had similar widths, but also had little variation in genomic positions. When the genomic conversion was to an older version of reference assembly of the same species (e.g. canfam3 to canfam2), query TADs recovered less well. Less than 10% of query TADs mapped uniquely to a single location in the target genome, although over 76% were intra-chromosomal splits. When genomic conversion was across species (hg19 to bostau6, mm9 to bostau6, mm10 to bostau7 and canfam2 to bostau6), over 98% of query TADs split and over 87% were inter-chromosomal splits. No input TADs were in chromosome Y or mitochondrial chromosome, but some TAD fragments were mapped to the bovine mitochondrial chromosome.

**Fig. 1:**
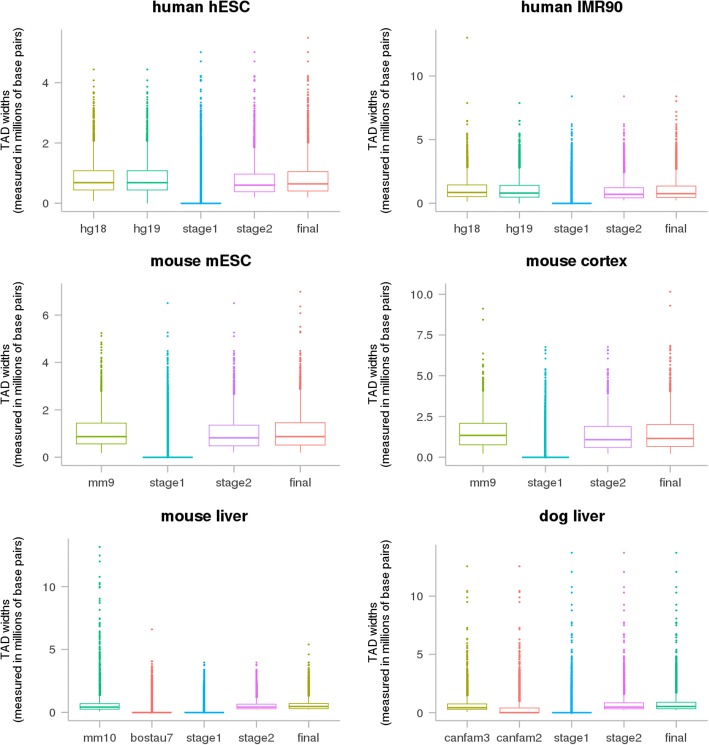
Distribution of TAD widths. For each input dataset, the TAD width (millions of base pairs) on input reference genome, intermediate reference genome (if it was used), bovine reference genome (putative bovine TADs stage 1, stage 2 and final) were shown as boxplots

To reduce the large number of bovine TAD fragments that were inevitably created during the mapping of large genomic segments, we used a recovery, a filtering and a local refinement procedure. Our method resulted in putative bovine TADs that resembled the respective input TADs in the following aspects: firstly, similar number of TADs were found in the bovine genome as the respective input TADs (Table 1); secondly, no putative bovine TADs aligned to chromosome Y or mitochondrial chromosome (Additional file 1: Table S1); thirdly, TAD widths were comparable between bovine and the source TADs (Fig. 1); finally, the majority of input TADs were mapped to the bovine genome (79.63–98.88%), and 65.55–89.03% of those mapped uniquely to a single genomic location without any intra-chromosomal or inter-chromosomal splits (Table 1). Putative bovine TAD genomic coordinates are provided as Additional file 2: Table S2.

**Table 1:** Summary statistics of TAD mapping

Input TADs		Reference assembly	Number of TADs	Mean TAD width (kb)	Number of input TADs mapped (ratio)		
Study	Cell or tissue				1 location in bovine genome	Same bovine chromosome	Bovine genome
Dixon 2012	hESC	hg18	3127	852.2	–	–	–
		bostau6	2885	830.5	2784(89.03%)	2930(93.70%)	2956(94.53%)
	IMR90	hg18	2349	1122	–	–	–
		bostau6	2236	1071	2050(87.27%)	2198(93.57%)	2261(96.25%)
	mESC	mm9	2200	1093	–	–	–
		bostau6	2173	1104	1912(86.91%)	2041(92.77%)	2127(96.68%)
	cortex	mm9	1519	1542	–	–	–
		bostau6	1597	1489	1283(84.46%)	1401(92.23%)	1502(98.88%)
Rudan 2015	liver	mm10	3643	695	–	–	–
		bostau6	3507	602.5	2388(65.55%)	2873(78.86%)	2901(79.63%)
		canfam3	3315	686.5	–	–	–
		bostau6	2979	810	2731(82.38%)	2887(87.09%)	2916(87.96%)

The source study and cell/tissue from input TAD dataset, and also the input and output reference assemblies with the number of TADs and their mean width (in kilobases) are shown. Also presented are the number of input TADs that did not split during mapping (1 location in the bovine genome), the number of input TADs that did not split or split intra-chromosomally during mapping (same bovine chromosome), and the number of input TADs that did not split, split intra-chromosomally or inter-chromosomally during mapping (bovine genome)

### Scanning for putative bovine liver CTCF binding motifs

Chromatin immunoprecipitation with sequencing (ChIP-Seq) is an assay that identifies where a protein and DNA interact in the sample. DNA sequence motifs from ChIP-Seq may correspond to the conserved units of protein-binding sites [27]. We downloaded 184,492 CTCF ChIP-Seq data from the liver tissue of human, mouse, dog and macaque [25] (Table 2). We identified 82 motif profiles (e-value ≤ 10^−5^) from these CTCF ChIP-Seq data. Of those, 4 profiles were validated in the JASPAR, UniProt or HOCOMOCO databases (Additional file 3: Appendix 1), and were also widely reported in literature [16, 24–26, 28–30]. All 82 motif profiles were scanned across bovine chromosome 1 to X, and 3,770,311 putative bovine CTCF binding motifs were found (*P*-value ≤ 10^−5^; Additional file 3: Appendix 1). Note that motifs on the same genomic coordinates but different DNA strands would be counted as 2 motifs. The putative bovine CTCF binding motifs were short (7-29 nt) and tended to group into clusters distributing sparsely across the entire bovine genome. Less than 14% putative bovine CTCF binding motifs overlapped with another putative bovine CTCF binding motif. We defined a more stringent set of CTCF binding motifs as those whose motif score was no less than 80, motif P-value was no larger than 10^−8^, and any overlapping regions on the same DNA strand were merged. We found 78,524 more stringent CTCF binding motifs on the bovine genome (Table 2). We defined CTCF gaps as the genomic intervals between the more stringent CTCF binding motifs. There were 45,809 CTCF gaps ranging from 1 bp to 1,503,697 bp.

**Table 2:** Summary statistics of identifying CTCF binding motifs in the bovine genome

Mammalian CTCF ChIP-Seq sequence			Putative bovine CTCF binding motifs (strand-specific)			
Quantity	Width (nt)	Number of motif profiles identified (e ≤ 10^−5^)	Set	Quantity	Width (nt)	make up % of bovine genome
184,492	42–1716	81	Less stringent	3,770,311	7–29	0.61%
			More stringent	78,524	15–97	0.02%

The number of input mammalian CTCF ChIP-Seq sequences and their range of lengths are shown. Also presented is the number of CTCF binding motif clusters identified from MEME-ChIP. Last presented are FIMO results that are categorised into two sets by filtering stringencies. A less stringent set of putative bovine CTCF binding motifs has motif P-value no larger than 10^−5^. A more stringent set of putative bovine CTCF binding motifs has motif *P*-value no larger than 10^−8^, motif score no smaller than 80, and non-overlapping. For each set the number of CTCF binding motifs discovered in the bovine genome, their range of lengths, and the proportion of these motifs making up the bovine genome are shown

### Enrichment of biological hallmarks at TAD boundaries

**Fig. 2:**
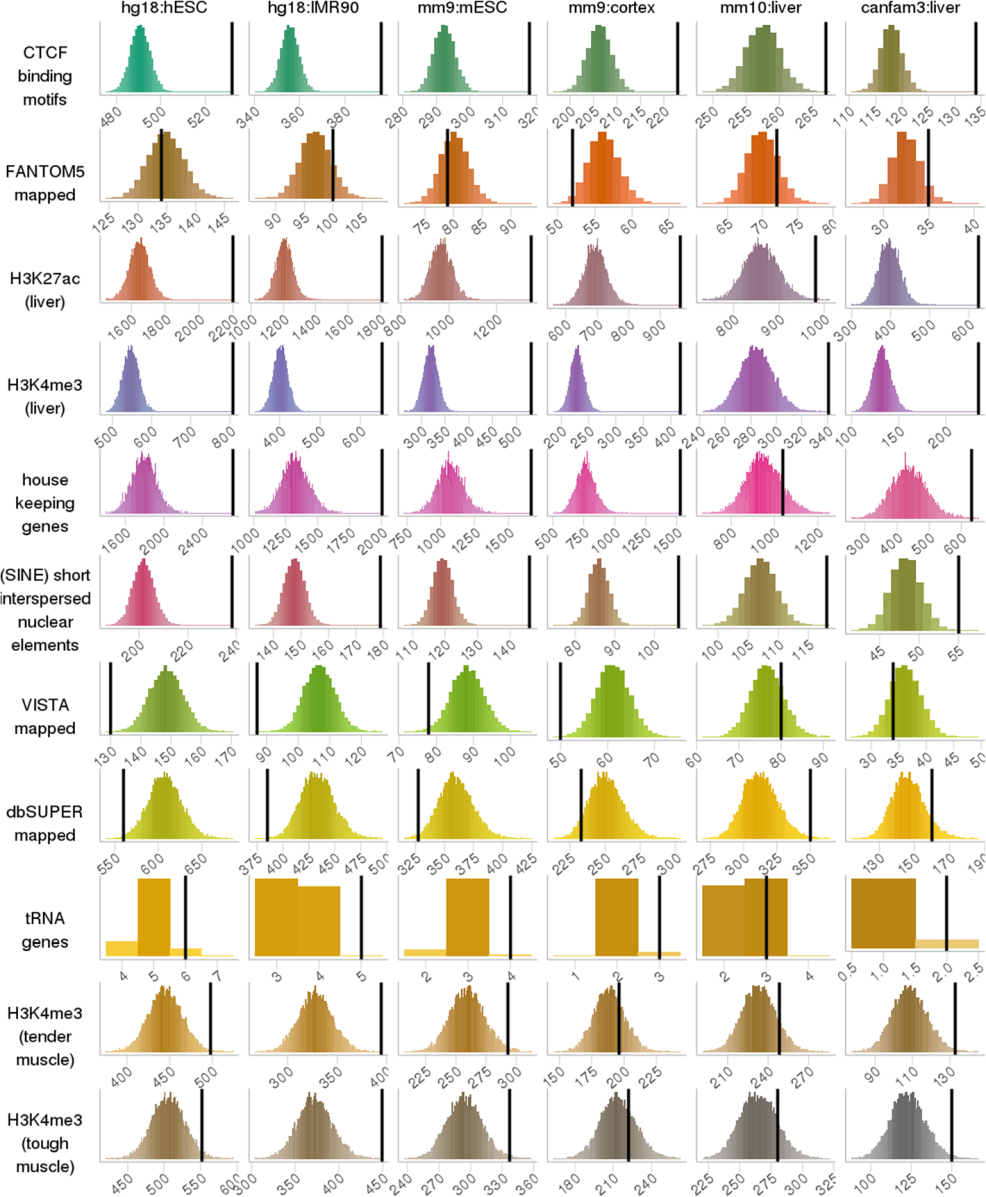
Enrichment of biological hallmarks in putative bovine TAD boundaries. For each hallmark biological signal (rows) and each putative bovine TAD set (columns), these frequency histograms show the number of overlapping base pairs between the signal and the TAD boundaries. The frequencies were rounded to the nearest 10 thousand, and the bin width was 1. The 10,000 random permutations are in colour and the actual number is the black vertical line. If a biological signal is enriched at TAD boundaries, the vertical line will be on the right and clearly separated from the histogram

We validated final sets of putative bovine TADs by assessing the level of enrichment of biological hallmarks at putative bovine TAD boundaries (Fig. 2; Additional file 4: Table S3A). The less stringent set of putative bovine CTCF binding, bovine liver H3K27ac and H3K4me3, bovine SINE and tRNA genes were all highly enriched in all sets of putative bovine TAD boundaries. House-keeping genes were highly enriched in all sets of putative bovine TAD boundaries but less so in putative bovine TAD set mapped from mouse liver. Bovine H3K4me3 from tender and tough muscle tissues were highly enriched in all sets of putative bovine TAD boundaries but less so in putative bovine TAD sets mapped from mouse cortex and liver. Putative bovine enhancers in homology with VISTA, FANTOM5 and dbSUPER datasets were not enriched in all sets of putative bovine TAD boundaries. The same permutation test was repeated but excluding ‘knots’, which meant that we assumed there were no TAD boundaries if the end position of the previous TAD was the same as the start position of the following. We found a very similar enrichment profile as those including knots (Additional file 4: Table S3A). The same permutation test was also repeated using the more stringent set of CTCF binding motifs, but we did not observe significant enrichment of the more stringent CTCF binding motifs at TAD boundaries across all TAD sets except at putative bovine TAD boundaries that were mapped from mouse liver (Additional file 4: Table S3A).

The putative bovine CTCF binding motifs had 108 patterns (Additional file 3: Appendix 1). To examine which patterns were enriched at TAD boundaries, we repeated the same enrichment analysis for each type of CTCF binding motif (Additional file 4: Table S3B). We found that patterns from the four putative CTCF binding motif profiles, which were validated in public databases were all highly enriched at all sets of putative bovine TAD boundaries (Additional file 3: Appendix 1; Additional file 4: Table S3). Some putative bovine CTCF binding motifs, even though were not validated by public TF binding motifs databases, were highly enriched at all sets of TAD boundaries (e.g. motif number 15 and 77 in Additional file 4: Table S3B). Some putative bovine CTCF binding motifs were only enriched at putative bovine TAD boundaries mapped from cell lines but were not as enriched at those mapped from tissues (e.g. motif number 6 and 11 in Additional file 4: Table S3B). A large number of CTCF binding motifs from the less stringent set were absent in the more stringent set, but only a few of those less stringent putative bovine CTCF binding motifs were enriched at TAD boundaries (e.g. motif number 52 and 77 in Additional file 4: Table S3B).

### Testing allele-specific expression within regulatory units

**Fig. 3:**
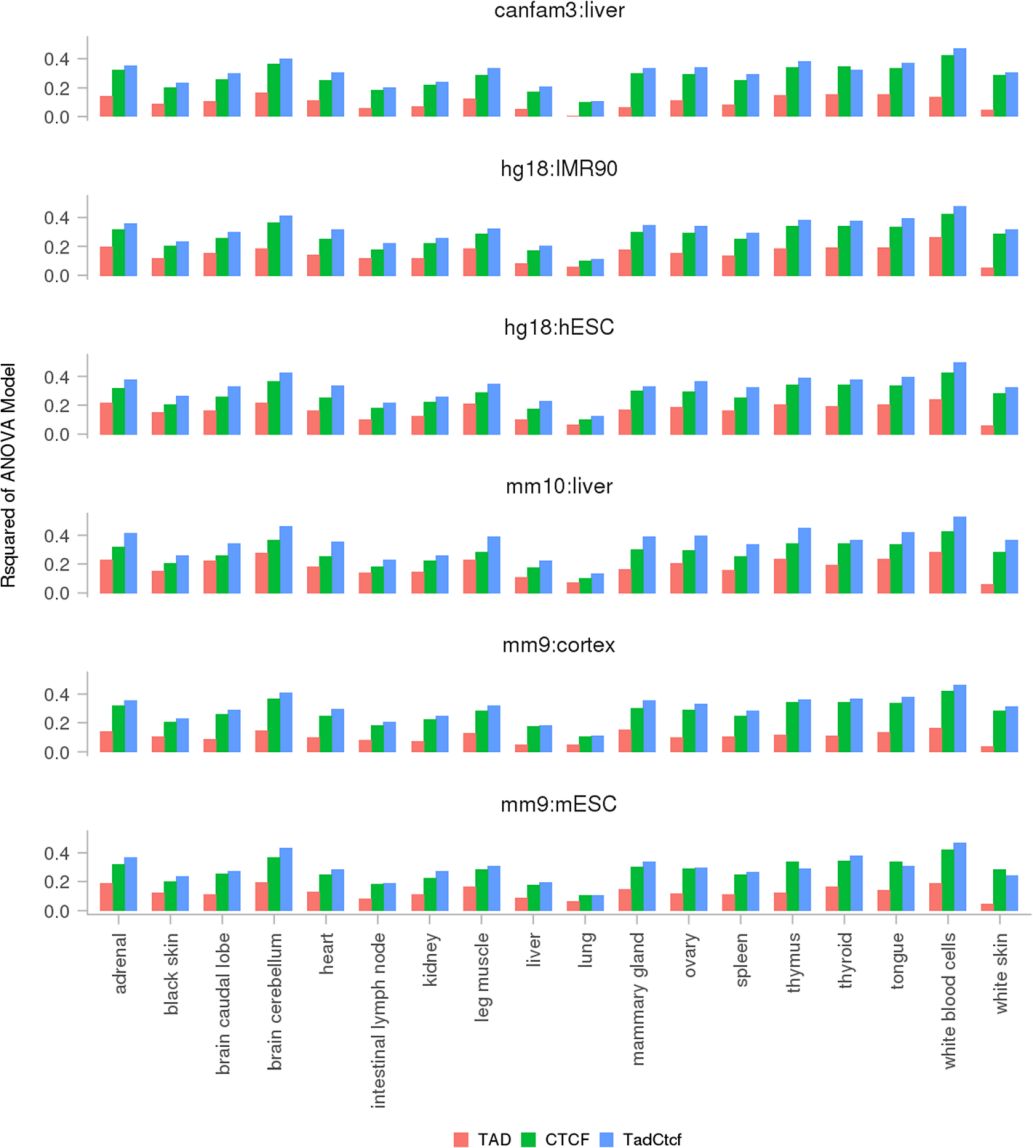
R-squared values from significant ANOVA models (P ≤ 10^−8^). For each tissue (X-axis) and each regulatory unit defined by TAD, CTCF gaps and TAD+CTCF gaps, these bar plots show the R-squared values of significant (P ≤ 10^−8^) ANOVA models (Y-axis). Note in the CTCF model, there is no input TAD set involved. For the purpose of a convenient comparison with the other models, the same R-squared values in the CTCF model were plotted across the same tissue

Runs of genes were found to favour the same parental allele in 1 lactating cow’s 18 tissues [31]. We used analysis of variance (ANOVA) to test whether exons within any regulatory units were significantly biased for expressing from a parental chromosome in a tissue. The regulatory units were respectively defined only by TAD, only by the more stringent set of CTCF binding motifs, and by both TAD and more stringent set of CTCF binding motifs in three analysis of variance (ANOVA) models. Of those 108 cohorts (6 TAD sets × 18 tissues) tested in the TAD only model, we found that on average 14% of allele-specific expression (ASE) variation were explained, with a standard deviation (SD) of 0.056 across 107 significant cohorts (P ≤ 10^−8^). The remaining cohort, putative bovine TAD set from dog liver, did not show significant ASE variation in lung. The putative bovine TADs from the mouse cortex explained the least amount of ASE variation in white skin (3.45%), and those from mouse liver explained the largest amount of ASE variation in white blood cells (28%; Fig. 3). The CTCF only model had 6% less ASE SNPs than the TAD only model had, and explained on average 27% (SD: 0.078) ASE variation across all 18 significant cohorts (1 CTCF gaps × 18 ASE tissues; P ≤ 10^−8^). The CTCF only model explained ASE variation across 18 tissues in a trend that was similar to the TAD only model, where the least amount of ASE variation explained was in lung (10%) followed by liver (17%), and the largest amount of ASE variation explained was in white blood cells (42%; Fig. 3). The TAD+CTCF model tested around 30% less ASE SNPs than the TAD only model, and explained on average 31% (SD: 0.089) ASE variation across all 108 significant cohorts (6 TAD sets × 18 tissues; P ≤ 10^−8^). Trend was also similar to the TAD only model and CTCF only model, where the least amount of ASE variation explained was also in lung (on average 11%; SD: 0.012), and the largest amount of ASE variation explained was also in white blood cells (on average 48%; SD: 0.023; Fig. 3). We found little increments, and sometimes even a slight decrease, in variation explained by the TAD+CTCF model in comparison to the CTCF only model, which indicated that the CTCF model encompassed the TAD model, and was better at explaining ASE variation. Our permutation tests declared strong significance for all cohorts in all models tested, meaning that our observation that the confinement of ASE variation within regulatory units was not random.

### Enrichment of significant aseQTLs and eQTLs within putative bovine CTCF binding motifs

**Fig. 4:**
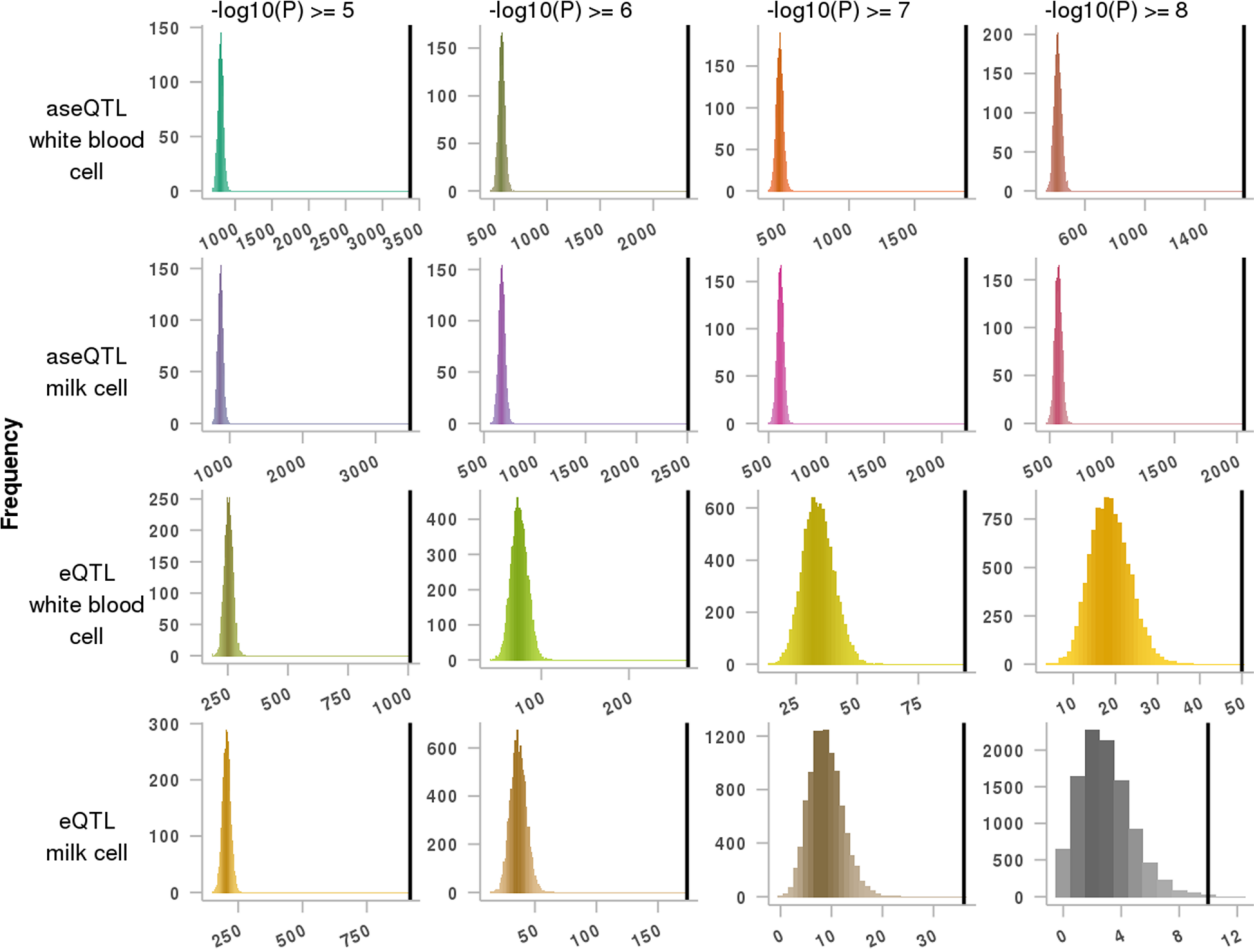
Enrichment of significant aseQTLs and eQTLs in putative bovine CTCF binding motifs. For each significant threshold (columns) and each aseQTL/eQTL in white blood cells or milk cells (rows), these frequency histograms show the number of significant aseQTLs/eQTLs in putative bovine CTCF binding motifs (motif score ≥ 80 and motif *P*-values ≤ 10^−8^). The 10,000 random permutations are in colour and the actual number is the black vertical line. If the actual significant aseQTLs/eQTLs are enriched at putative bovine CTCF binding motifs, the vertical line will be on the right and clearly separated from the histogram

We defined significant aseQTLs and eQTLs by the *P*-value from aseQTL and eQTL mappings respectively [32, 33]. We found across all P-value thresholds tested ranging from 10^−5^ to 10^−8^, the significant aseQTLs and eQTLs in white blood and milk cell were highly enriched at putative bovine CTCF binding motifs (P-value ≤ 10^−5^; Table 3) in comparison to the null distribution sampled from the entire bovine genome. The averaged fold change of enrichment for significant aseQTLs in white blood cells was 1.55 fold and in milk cells was 1.49 fold. The averaged fold change of enrichment for significant eQTLs in white blood cell was 1.43 fold and in milk cells was 1.41 fold (Table 3). The same permutation test was repeated in the more stringent set of CTCF binding motifs (motif score ≥ 80 and motif *P*-value ≤10^−8^), and significant aseQTLs and eQTLs in white blood and milk cell were also highly enriched across all P-value thresholds tested (Table 3; Fig. 4). The fold change of enrichment was higher in the more stringent set of CTCF binding motifs. On average, the significant aseQTLs in white blood cells were 4.06 fold and in milk cells were 3.74 fold, and the significant eQTLs in white blood cells were 3.23 fold and in milk cells were 4.18 fold more enriched in comparison to the rest of the genome (Table 3; Fig. 4). The same method was also used to test the level of enrichment for significant aseQTLs and eQTLs in biological hallmarks including H3K4me3 regions from both bovine liver and muscle tissues, and H3K27ac regions from bovine liver tissue, and bovine genomic regions in homology with VISTA, FANTOM5 and dbSUPER databases. We found that across all *P*-value thresholds tested, significant aseQTLs and eQTLs were less enriched in bovine specific H3K4me3 and H3K27ac regions than in putative bovine CTCF binding motifs, and significant aseQTLs and eQTLs were depleted in bovine genomic regions in homology with VISTA, FANTOM5 and dbSUPER databases (Additional file 5: Table S4).

**Table 3:** Rank and fold change of enrichment of significant aseQTLs and eQTLs at putative bovine CTCF binding motifs

QTL type	cell type	P-value significant threshold	Less stringent		More stringent	
			rank	fold change	rank	fold change
aseQTL	white blood	10^−5^	<0.0001	1.54	<0.0001	4.19
		10^−6^	<0.0001	1.55	<0.0001	4.07
		10^−7^	<0.0001	1.55	<0.0001	4.02
		10^−8^	<0.0001	1.56	<0.0001	3.96
	milk	10^−5^	<0.0001	1.49	<0.0001	3.97
		10^−6^	<0.0001	1.49	<0.0001	3.71
		10^−7^	<0.0001	1.49	<0.0001	3.67
		10^−8^	<0.0001	1.48	<0.0001	3.61
eQTL	white blood	10^−5^	<0.0001	1.38	<0.0001	3.97
		10^−6^	<0.0001	1.43	<0.0001	3.56
		10^−7^	<0.0001	1.45	<0.0001	2.75
		10^−8^	<0.0001	1.47	<0.0001	2.66
	milk	10^−5^	<0.0001	1.34	<0.0001	4.48
		10^−6^	<0.0001	1.40	<0.0001	4.75
		10^−7^	<0.0001	1.38	<0.0001	4.05
		10^−8^	<0.0001	1.53	0.9978	3.44

The levels of enrichment of significant allele-specific expression quantitative trait loci (aseQTL) and expression quantitative trait loci (eQTL) from bovine white blood cells and milk cells at two sets of putative bovine CTCF binding motifs are presented. The significance is defined by a P-value less than 10^−5^, 10^−6^, 10^−7^and 10^−8^ in aseQTL mapping and eQTL mapping respectively. A less stringent set of putative bovine CTCF binding motifs has motif P-value no larger than 10^−5^. A more stringent set of putative bovine CTCF binding motifs has motif P-value no larger than 10^−8^ and motif score no smaller than 80

### Testing frequency of each eGene and its most significant *cis-*QTL locating within the same TAD

TADs are evolutionarily conserved regulatory units that partition the genome by self-interaction frequencies [8, 14–16]. To assess whether our putative bovine TADs could also partition the bovine genome by self-interaction frequencies, we tested the frequency of each eGene and its most significant *cis-*QTL (i.e. aseQTL and eQTL) being within the same TAD. We required the most significant aseQTL and eQTL for each eGene to pass a P-value threshold, which was tested from 10^−5^ to 10^−8^. When multiple aseQTLs or eQTLs were ranked the highest significance towards the same eGene, we selected linearly the furthermost aseQTL and eQTL for each eGene in order to avoid bias favouring the aseQTL and eQTL in a linearly closer distance to the eGene. Across all P-value thresholds tested and in both white blood and milk cell samples, the observed numbers of furthermost, and most-significant, aseQTLs and eQTLs within the same TAD as their eGene were all significantly more than expected, with the Chi-Squared test *P*-values all close to 0 (Table 4; Additional file 6: Table S5). This indicated that our putative bovine TADs could provide the right space to search for *cis*-regulatory variants and their genic targets.

**Table 4:** eGenes are often located within the same TAD as their linearly-furthermost, and most-significant, aseQTL/eQTL

QTL type	Cell type	maximum number of eSNPs tested in a chromosome	Sourced TAD set	number of significant eGene in TAD	number of furthermost and most-significant *cis-*QTL-eGene in the same TAD		Chi-Square test	
					Expected	observed	value	*P*-value
aseQTL	white blood	1,057,269	hg18:hESC	13,302	1406.379224	6375	17,553.7238	0
			hg18:IMR90	13,790	1457.973951	7273	23,192.8204	0
			mm9:mESC	13,641	1442.220643	7682	26,996.4562	0
			mm9:cortex	13,150	1390.308735	8670	38,116.6453	0
			mm10:liver	12,013	1270.09725	5101	11,554.8757	0
			canfam3:liver	13,046	1379.313137	6086	16,060.8209	0
	milk	1,153,815	hg18:hESC	9553	1102.23947	3862	6909.82168	0
			hg18:IMR90	10,019	1156.007249	4550	9964.63198	0
			mm9:mESC	9706	1119.892839	4764	11,857.8462	0
			mm9:cortex	9317	1075.009436	5608	19,114.2541	0
			mm10:liver	8692	1002.895998	2931	3706.85001	0
			canfam3:liver	9638	1112.046897	3863	6805.23726	0
eQTL	white blood	8964	hg18:hESC	327	0.2931228	114	44,108.66	0
			hg18:IMR90	340	0.304776	125	51,017.4649	0
			mm9:mESC	322	0.2886408	125	53,883.3149	0
			mm9:cortex	305	0.273402	123	55,090.3719	0
			mm10:liver	284	0.2545776	91	32,346.6467	0
			canfam3:liver	330	0.295812	119	47,633.917	0
	milk	2463	hg18:hESC	343	0.0844809	24	6770.19366	0
			hg18:IMR90	348	0.0857124	25	7241.9128	0
			mm9:mESC	356	0.0876828	34	13,115.9732	0
			mm9:cortex	323	0.0795549	36	16,218.7166	0
			mm10:liver	340	0.083742	23	6271.10507	0
			canfam3:liver	362	0.0891606	22	5384.49588	0

The linearly-furthermost, and most-significant, aseQTL and eQTL for each eGene all have a *P*-value less than 10^−8^ in aseQTL/eQTL mapping

## Discussion

We have presented a computational method that identifies topological association domains (TADs) based on sequence homology. Our method relies on the conserved nature of TADs and the quality of reference genome assemblies. A small proportion (1–21%) of mammalian TADs were filtered out in the final bovine TAD sets (Additional file 1: Table S1), because they did not fulfil the size (>200Kb) or the consensus (> 3 TAD sets agreed) requirement. Overall, input TADs were mapped to similar locations in the bovine genome, and mapped bovine TADs displayed similar features of TAD widths and boundary signals as input TADs from Hi-C data (Figs. 1 and 2; Additional file 1: Table S1). These indicated that most input TADs were intact in the bovine genome, and our mapped TADs were a good proxy for the actual bovine TADs.

We have also created a library of putative bovine CTCF binding motifs from mammalian CTCF ChIP-Seq data. Only the highly conserved CTCF binding motifs were validated by TF binding motifs that were made available to public databases (Additional file 3: Appendix 1), and these conserved putative bovine CTCF binding motifs were enriched in all sets of putative bovine TAD boundaries (Additional file 4: Table S3). Different transcription factor binding motif (TFBM) databases have validated different CTCF binding motifs, and some non-validated CTCF binding motifs were also enriched at all sets of putative bovine TAD boundaries. These indicated that the current CTCF binding motifs in public TFBM databases were incomplete, and called for implementing projects such as the Functional Annotation of Animal Genomes (FAANG) [34] to generate bovine specific CTCF-ChIP data.

We created two sets of putative bovine CTCF binding motifs, which were differed by *P*-value stringencies. The less stringent set (*P*-value ≤ 10^−5^) were more enriched at TAD boundaries than the more stringent set (motif score ≥ 80, motif *P*-value ≤ 10^−8^, non-overlapping). One explanation for this observation is that the more stringent set were too sparsely distributed across the entire bovine genome and therefore failed an enrichment analysis. We observed where the more stringent CTCF binding motifs were found, similar motif patterns were also found in the less conserved set (Additional file 4: Table S3B), which implied the same genomic locations tagged.

TADs are defined from Hi-C data which snapshots chromosomal regions in close spatial proximities without specifying any target loci [8, 10]. Contact maps similar to TADs can also be constructed from chromatin interaction analysis by pair-end tag sequencing (ChIA-PET), which snapshots chromosomal regions in close spatial proximities mediated by a specific factor [35]. Interestingly, chromosomal contact domains identified by CTCF ChIA-PET data were found to be highly assembled to the TADs identified from Hi-C data [36]. This inspired us to investigate whether the overall direction of ASE (paternal or maternal) were confined within putative bovine TADs and between CTCF binding motifs. We acknowledge that we did not explicitly  test whether the individual SNP within the same gene support the same direction of ASE, but rather that the overall effect on ASE across genes was in the same direction. We found the overall direction of ASE within TADs and between CTCF binding motifs were confined across 18 bovine tissues. The effects of TAD and CTCF on ASE variation remained as significant when gene was considered in the ANOVA model (Additional file 7: Appendix 2), indicating that TAD and CTCF were independent of genes contributing to ASE variation.

We found CTCF binding motifs were better than TADs at predicting ASE variation. To illustrate this, we showed a region on bovine chromosome 3 spanning from position 54 million to position 55.2 million (Additional file 8: Figure S1A). All our bovine TAD sets from Dixon et al. [14] predicted this region as a putative bovine TAD, and several CTCF binding motifs were found within this region. All three ANOVA models found this region displayed significant ASE. Inside this region, *GBP5* (ENSBTAG00000015060), *ENSBTAG00000017670* and *ENSBTAG00000038938* genes were biased towards maternal expression in white blood cells [31], and *ENSBTAG00000014857*, *ENSBTAG00000037490* and *ENSBTAG00000038500* genes were slightly paternally biased. A cluster of SNPs inside *GBP5*, *ENSBTAG00000017670* and *ENSBTAG00000038938* genes were maternally biased. As a result, both TAD-only and CTCF-only ANOVA models found this region maternally biased (for this animal at least). Interestingly, *GBP5*, *ENSBTAG00000017670* and *ENSBTAG00000038938* genes were also located between CTCF binding motifs. As a result, TAD+CTCF model found that the genomic regions between CTCF binding motifs maternally biased, and the remaining regions paternally biased (Additional file 8: Figure S1). Another example is provided in Additional file 8: Figure S1B.

One explanation why CTCF was better than TAD at predicting ASE regions is that CTCF not only mediated the sub-TAD structures [15, 36], but also harboured *cis-*regulatory variants [37–39]. To test this hypothesis, we examined whether significant aseQTLs and eQTLs from bovine white blood and milk cells were enriched at putative bovine CTCF binding motifs. We found even though bovine aseQTLs and eQTLs were from a different cell type from putative bovine CTCF binding motifs, significant aseQTLs and eQTLs were still close to 4-fold enriched at the more stringent set of putative bovine CTCF binding motifs (Table 3; Fig. 4), and were more enriched at putative bovine CTCF binding motifs than at any other biological signals tested (Additional file 5: Table S4). These indicated that CTCF binding motifs that were leveraged through homology are informative for annotating the bovine genome.

Regulatory units are used as a scaffold for testing long-range association or supporting molecular QTLs discovered in humans [36, 40]. To assess whether our predicted bovine TADs have the potential to reduce the space for causative regulatory variants to sites of physical proximity, we examined whether or not each eGene and its most significant aseQTL and eQTL were often located in the same TAD. Our result showed that the linearly-furthermost, and most-significant, white blood and milk cells aseQTL and eQTL were both more likely to be within the same TAD as the eGene than not (Table 4; Additional file 5: Table S5). This indicated that our results could be very useful for reducing the search space for causative regulatory variants and their eGenes. Without knowing the regulatory units, an aseQTL study [32] had to perform 4.8 billion association tests for 291,638 genic positions and all SNPs (as identified by the 1000 Bull Genomes Project [1]) within ±1 Mb to those genes, and an eQTL study [32] had to perform 83 million association tests for 11,577 genes and all SNPs within ±1 Mb to those genes. If regulatory units were known, the number of association tests could be largely reduced to only test those that were within the same regulatory unit, independent of the distance between the heterozygous SNP and the gene. The number of tests could continue to be reduced by testing associations only between heterozygous SNPs in putative regulatory regions such as enhancers, promoters and CTCF binding motifs. Another benefit of annotating functional regions such as TADs and CTCF binding motifs is that new methods that aim to improve the accuracy and reliability of genomic prediction can be devised. Existing methods, such as MultiBLUP [41] and BayesRC [42], could demonstrate higher power by assigning different distributions of effects to different regulatory annotations. This would help prioritise ‘lead’ SNPs for genomic prediction [1].

## Conclusions

In conclusion, our study shows that homologous topological association domains (TADs) and CTCF binding motifs can reduce the search space for causative regulatory variants in the bovine genome. Along with study that predict functional elements such as enhancers and promoters across species [43], these results complement the experimental validation that the Functional Annotation of Animal Genomes (FAANG) consortium [34] will produce, and accelerate the identification of mutations that affect complex traits in the bovine genome.

## Methods

### Mapping mammalian topological association domains to the bovine genome

Topological association domain (TAD) genomic coordinates from human and mouse embryonic stem cells (hESCs and mESCs) [14], human IMR90 fibroblasts (IMR90) [14] and mouse cortex [44] were obtained from Yue Lab (http://promoter.bx.psu.edu/hi-c/download.html). TAD genomic coordinates from the fresh or frozen liver of mouse and dog were provided as supplementary Additional file 1: Table S1 by Rudan et al. [16]. Genomic coordinate conversion files, mammalian reference genomes and mammalian genome annotations were all loaded through Bioconductor (version 3.2) [45].

All genomic coordinates of mammalian TADs were converted to the bovine reference genome *Bos taurus* UMD3.1 (UCSC Genome Browser assembly ID: bostau6) using UCSC Batch Coordinate Conversion program (liftOver) [46] (default settings), which was run inside R (version 3.2.4) using Bioconductor (version 3.2) [45] (AnnotationHub [47], rtracklayer [48] and GenomicRanges [49] packages). Genomic coordinate conversion files were required as an input to liftOver, but were absent for the conversion of some versions of reference genome assemblies. In those cases, an intermediate genome was used to convert the source TADs to bostau6. As a result, the hESC and IMR90 TADs were mapped from hg18 to bostau6 in two steps through an hg19 intermediate. The mESC and mouse cortex TADs were mapped directly to bostau6. The mouse liver TADs were mapped from mm10 to bostau6 through bostau7 (NCBI ID: Btau4.6.1.). The dog liver TADs were mapped to bostau6 from canfam3 through canfam2 (Additional file 9: Figure S2).

Converting large genomic segments inevitably created a large number of genomic fragments. We employed a recovery procedure, a filtering procedure, and a local refinement procedure to finalise the putative bovine TADs. Throughout this study, we specified “input” as the data that was input to the first step of the genomic conversion, “query” as the data that was input to each further step of the genomic conversion, and “target” as the output from each step of the genomic conversion. The recovery procedure was employed in each step of the genomic conversion as follows:

For each input TAD set, *w* was denoted as the minimum width of input TADs.

1. If TAD fragments from the same query sequence were no more than *w* away from each other in the target genome, the TAD fragments were merged into a single TAD in the target genome.
2. If TAD fragments from different query sequences overlapped in the target genome, the TAD fragments were kept as separated fragments in the target genome.

Genomic conversion in combination with the recovery procedure resulted in 6 sets of putative bovine TADs (stage 1) that were respectively from the 6 sets of input TADs. A filtering procedure was then applied to the putative bovine TADs (stage 1), in order to reduce TAD fragments of low confidence in each TAD set. The filtering procedure was performed as follows:

1. For each genomic position *i* in the bovine genome, the number of TAD sets that agreed *i* was in a TAD was calculated and denoted as *a*_*i*_.
2. If a putative bovine TAD (stage 1) was no less than 200 Kb wide, and contained at least one position *i* where *a*_*i*_ was no less than 4, this TAD (stage 1) was kept in the TAD set (stage 2). The 200-Kb threshold was selected because it was able to effectively filter out the small homological genomic fragments which were incapable of forming a TAD in the bovine genome.

The filtering procedure resulted in 6 sets of putative bovine TADs (stage 2) that were respectively from the 6 sets of putative bovine TADs (stage 1). A local refinement procedure was then applied to merge any putative bovine TADs (stage 2) that overlapped more than 1 bp. The 1 bp threshold (exclusively) was used, because TADs were calculated from frequencies of interactions between genomic bins [14, 16]. This resulted in the end genomic coordinates of some TADs being the same as the start genomic coordinates of their downstream TAD. These ‘TAD1.TAD2’ structures were shown in the input TAD sets, and if no large genomic rearrangements occurred, the homologous counterparts of these ‘TAD1.TAD2’ structure were kept in the final bovine TAD set. However, when large genomic re-arrangements occurred, source TADs from the same TAD set could overlap to a great extent in the bovine genome. Each of these overlapping TAD fragments was > 200 Kb with high frequency of internal interactions, indicating that they could form into a larger interaction domain in the bovine genome, and therefore were merged into one putative bovine TAD. The local refinement procedure resulted in 6 sets of final bovine TADs that were respectively from the 6 sets of putative bovine TADs (stage 2). A schematic workflow of the TAD mapping procedure is provided as Additional file 9: Figure S2.

### Scanning for putative bovine liver CTCF binding motifs

CTCF binding genomic coordinates from the liver tissue of human, mouse, dog and macaque were downloaded from E-MTAB-437 [25]. Mammalian CTCF binding sequences were extracted from masked reference genome hg19, mm9, canfam2 and rhemac2 respectively. Masked reference genome is reference genome where interspersed repeats and low complexity DNA regions are detected and concealed, and is required for motif discovery.

All mammalian CTCF binding sequences were provided as one input to MEME-ChIP [50] in order to identify evolutionarily conserved CTCF binding motifs. Parameter settings for MEME-ChIP were based on the MEME program settings in Vietri Rudan et al. [16], who also used the same CTCF ChIP-Seq data from Schmidt et al. [25]. We looked for 0 or 1 DNA motif per ChIP-Seq peak, and the maximum input dataset size was adjusted to 70 Mb to account for our input file size.

The latest version of JASPAR CORE motif databases (2016), JASPAR CNE motif database (2008), JASPAR POLII database (2008), JASPAR PHYLOFACTS (2008), UniPROBE [51], and *Homo sapiens* comprehensive model collection database (HOCOMOCO; version 10) were obtained from MEME Suite [27] (http://meme-suite.org/). These TF binding motifs that were made available to public databases were used to validate our CTCF binding motif profiles from MEME-ChIP outputs.

Since only the more conserved CTCF binding motifs are reported to TF binding motifs databases, and an increasing number of novel motifs are discovered as more CTCF ChIP-Seq data are made available, all motifs from MEME-ChIP were input to FIMO [52] (default settings) to scan for the occurrence of putative CTCF binding motifs in the bovine genome. We created two sets of putative bovine CTCF binding motifs from FIMO outputs which differed by *P*-value stringencies. A less stringent set of putative bovine CTCF binding motifs was selected by a motif P-value no larger than 10^−5^. A more stringent set of putative bovine CTCF binding motifs was selected by a motif score no less than 80 and a motif *P*-value no larger than 10^−8^ and where overlapping regions were merged. Both sets of putative bovine CTCF binding motifs were used for all downstream analyses in this paper unless otherwise noted.

### Enrichment of biological hallmarks at topological association domain boundaries

Bovine transfer RNA (tRNA) genes and short interspersed element (SINE) annotations were all loaded through Bioconductor (version 3.2) [45]. Human housekeeping gene names [53] were downloaded as per paper and were used as the list of bovine housekeeping genes. Bovine genomic regions that were enriched for tri-methylation of lysine 4 on histone H3 (H3K4me3) and acetylated lysine 27 on histone H3 (H3K27ac) signals from bovine liver tissue were downloaded from E-MTAB-2633 [54]. Bovine genomic regions that were enriched for H3K4me3 signal from the tender and tough longissimus dorsi of Aberdeen Angus steers were downloaded from GSE61936 [55]. Putative bovine enhancers in homology with human and mouse enhancer databases from VISTA, FANTOM5 and dbSUPER were as described by Wang et al. [56].

Putative bovine TAD boundaries were defined as those genomic intervals, no larger than 400 Kb, between adjacent putative bovine TADs from the same TAD set. For those putative bovine TADs where the end position of the previous TAD was the same as the start position of the following (i.e. a knot), 400-Kb genomic intervals centring on the “knot” were selected as TAD boundaries. A schematic graphical representation of the TAD, TAD boundaries and ‘knot’ is provided as Additional file 10: Figure S3. The level of enrichment of the following biological hallmarks was tested at TAD boundaries from each set of putative bovine TADs: human housekeeping genes, bovine tRNA genes, bovine SINEs, putative bovine CTCF binding motifs, putative bovine enhancer regions in homology with those in VISTA, FANTOM5 and dbSUPER databases, bovine liver H3K4me3 regions, bovine liver H3K27ac regions, bovine tender and tough muscle H3K4me3 regions.

The enrichment analysis was run as a permutation test with 10,000 random repeats to test whether the biological signal overlapped significantly more with the putative bovine TAD boundaries than the rest of the genome. We denoted *n* as the number of base pairs of a biological signal that overlapped with the putative bovine TAD boundaries in the original dataset. In the permutation test, for each chromosome, we slid the TAD boundaries along the chromosome by *M* positions, where *M* was a number randomly selected between 1 and the length of the chromosome. After sliding, if a TAD boundary exceeded the range of the chromosome, we recycled the exceeding subset of the TAD boundaries to the start of the chromosome. Then we counted the number of base pairs of a biological signal overlapped with the slid putative bovine TAD boundaries and denoted this number as *m*. The fold change of enrichment was defined as the ratio of *n* to the mean of all *m* values in the 10,000 permutations. The ranking position of *n* within the distribution of all *m* values over all random samples, denoted as *R*, was determined, and a *P*-value to test the significance of the ranking was computed. For the largest *n* among all *m* values, the *P*-value was set to <0.0001 and otherwise it was $\frac{R}{10001}$. We declared a biological signal “highly enriched” in a set of putative bovine TAD boundaries if *n* was larger than 95% of all *m* values. Our permutation tests resulted in 66 independent analyses (6 sets of finalised putative bovine TADs × 11 biological signals).

### Testing allele-specific expression within regulatory units

Chamberlain et al. [31] observed runs of genes expressed in favour of the same parental chromosome. We argued that these runs of allele-specifically expressed genes could be located within the same TADs and or between the same CTCF binding motifs. To investigate this, we obtained allele-specific expression (ASE) data from Chamberlain et al. [31]. The ASE data was from 1 lactating cow’s 18 tissues. In each tissue, the ASE data was the number of RNA transcripts aligned to each parental allele at all heterozygous exonic positions. The heterozygous exonic positions were determined from the cow’s whole genome sequence variants [31], and mapping bias was taken into account following methods that were described by Degner et al. [57]. The ASE data was used to calculate ASE scores as follows:

1 $$ {Y}_i=\mathit{\log}10\ \left(\frac{P_i+1}{M_i+1}\right) $$

where *Y*_*i*_ was the ASE score from a tissue at position *i*; *P*_*i*_ was the number of RNA reads from a tissue aligning to position *i* that had the paternal allele; and *M*_*i*_ was the number of RNA reads from a tissue aligning to position *i* that had the maternal allele. One was added to all read counts in order to obtain valid ASE scores even for the mono-allelic expressed SNPs, i.e. where only one of the two alleles were expressed. A positive ASE score meant that more parental than maternal alleles were detected among RNA reads at position *i*. A negative ASE score meant that more maternal than parental alleles were detected among RNA reads at position *i*. An ASE score of zero meant that equalled numbers of parental and maternal alleles were detected among RNA reads at position *i*.

We found that most heterozygous exonic SNPs were distributed within regulatory units. If most heterozygous exonic SNPs in the same regulatory units were from the same parental chromosome, the mean ASE scores of those SNPs would be significantly deviated from 0; alternatively, if heterozygous exonic SNPs were randomly expressed from parental chromosomes, the mean ASE scores of those SNPs would be close to 0. Analyses of variance (ANOVA) models were used to quantify this ASE variation (measured by mean ASE scores) from regulatory unit to regulatory unit. Three independent ANOVA models were fitted, where the ASE scores in a tissue were fitted as the response in all three ANOVA models, but the regulatory units in each model were different:

- Model (1) was a TAD only model that defined a regulatory unit only by the putative bovine TADs from a TAD set.
- Model (2) was a CTCF only model that defined a regulatory unit only by the CTCF gaps, which were the genomic intervals between the set of 2 CTCF binding motifs (motif score ≥ 80 and motif *P*-value ≤ 10^−8^).
- Model (3) was a TAD+CTCF model that defined a regulatory unit by both TAD and CTCF gaps. The CTCF gaps were embedded within the putative bovine TADs from a TAD set. Model (3) tested if TAD or CTCF gaps accounted for more variation observed in the ASE data.

In each ANOVA model, all innermost genomic regions were required to contain no less than 5 SNPs that had valid ASE scores. We found majority of the valid ASE SNPs (> 94% in TAD only model, > 82% in CTCF only model, and > 73% in TAD+CTCF model) were distributed among different genes within the same regulatory unit. There were 108 independent ANOVA tests performed in model (1) which were from the combination of 6 sets of finalised putative bovine TADs and 18 bovine tissues, 18 independent ANOVA tests performed in model (2) from the combination of 1 set of CTCF gaps and 18 tissues, and 108 independent ANOVA tests in model (3) from the combination of 6 sets of finalised putative bovine TADs and 18 tissues. The significance of an ANOVA test was declared at *P*-value ≤ 10^−8^.

The permutation tests, with 10,000 repeats, were performed to test whether the observed ANOVA result in each model was random. In each permutation test, the ASE scores were shuffled across the whole genome and then the model was refitted with the permuted dataset. The R-squared value from the original dataset, *R*, was compared with the 10,000 null R-squared values from the random shuffles, *R*^′^. A significant ANOVA cohort was declared if *R* was larger than all *R*^′^ values. In a significant ANOVA test, a regulatory unit was declared to display significant ASE effects if the absolute value of the averaged ASE scores in the region, |*n*|, was larger than 99% of the absolute value of the averaged ASE scores in the permutations ∣*m*∣, i.e. false discovery rate (FDR) < 0.01, and the *p*-value for *n* was no larger than 10^−6^. There were 108 independent permutation tests performed in model (1), 18 permutation tests performed in model (2) and 108 permutation tests performed in model (3).

### Enrichment of significant aseQTLs and eQTLs within putative bovine CTCF binding motifs

The heterozygous quantitative trait loci (QTLs) that were associated with allelic-specific expression (aseQTLs) and expression variation (eQTLs) were obtained from Chamberlain et al. [32, 33]. The aseQTL and eQTL mappings were performed using mRNA transcripts from 141 lactating cows’ white blood and milk cells, and mapping bias was taken into account following the methods that were described by Chamberlain et al. [32]. The aseQTL and eQTL data was a table of effect and *P*-value between an aseQTL/eQTL and its eGene target. The RNA sequence data is available from NCBI Sequence Read Archive (Bioproject accession PRJNA305942).

We hypothesized that significant *cis-*expressed QTLs were enriched at putative bovine CTCF binding motifs. We tested this hypothesis using aseQTLs and eQTLs from white blood and milk cells, both of which indicate *cis-*expression QTLs. The significant *cis*-QTLs were defined by a P-value threshold *p*, which was tested at 10^−5^ to 10^−8^. In each significant threshold, we selected a type of *cis*-QTLs (e.g. aseQTLs from white blood cells) whose P-value was no larger than *p*. In the case where multiple eGenes were found to be significantly associated with the same *cis*-QTL, the *cis*-QTL was only counted once. Then we calculated the number of significant *cis*-QTLs within putative bovine CTCF binding motifs, and denoted this number as *n*.

To test the frequency of significant *cis*-QTLs occurring in the rest of the genome, for each chromosome, we slid the significant *cis*-QTLs by *M* positions, where *M* was a number randomly selected between 1 and the length of the chromosome. After sliding, if the position of a *cis*-QTL exceeded the range of the chromosome, we redefined the position of the *cis*-QTL as the slid position subtracting the chromosome length. We denoted *m* as the number of those slid *cis*-QTLs within putative bovine CTCF binding motifs. The slide and recalculation were repeated 10,000 times. The fold change of enrichment was defined as the ratio of *n* to the mean of all *m* values from the 10,000 permutations. The ranking position of *n* within the distribution of all *m* values, denoted as *R*, was determined, and a *P*-value to test the significance of the ranking was computed. For the largest *n* among all *m* values, the *P*-value was set to <0.0001 and otherwise it was $\frac{R}{10001}$. We declared significant *cis*-QTLs highly enriched at putative bovine CTCF binding motifs if *n* was larger than 99% of all *m* values (FDR < 0.01). Our tests resulted in 32 independent analyses (2 types of *cis*-QTLs × 2 cell types × 4 significant thresholds × 2 sets of CTCF binding motifs).

### Testing frequency of each eGene and its most significant *cis-*QTL locating within the same TAD

We hypothesized that the most significant *cis-*expressed QTLs for each eGene fell more often within the same TAD as the eGene than not. We tested this hypothesis using aseQTLs and eQTLs, both of which indicate *cis-*expression QTLs. Our *cis-*QTLs were detected using mRNA transcripts from 141 lactating cows’ white blood and milk cells [32]. We selected the most-significant *cis*-QTL for each eGene within a TAD. The most-significant *cis*-QTL for each eGene was required to pass a significance threshold which was tested from 10^−5^ to 10^−8^. In the case where multiple *cis*-QTLs were ranked with the most significance level towards the same eGene, we broke the tie by selecting the *cis*-QTL that was linearly the furthest away from the eGene. The linearly furthermost *cis*-QTL was chosen in order to avoid bias favouring the most significant *cis*-QTL to be within the same TAD as the eGene. The result of this procedure was a number of *cis*-QTL and eGene targets, which occurred in the same TAD out of all possible pairs. The expected number of significant aseQTL/eQTL and eGene within the same TAD was defined as follows:

2 $$ E=N\times \overline{M}\times p $$

where *E* is the expected number of significant *cis*-QTL and eGene within the same TAD, *N* is the total number of eGenes overlapping a TAD, and $\overline{M}$ is the maximum number of eSNPs in a chromosome that were used for the aseQTL/eQTL mapping, and *p* was the P-value threshold for the association between the eSNP and the eGene.

A chi-square test was performed to test whether the observed number of furthermost and most-significant *cis*-QTL and eGene within the same TAD was statistically significant or not. The chi-square test was defined as follows:

3 $$ {\chi}^2=\frac{{\left(E-O\right)}^2}{E} $$

where *χ*^2^ is the chi-squared value, *E* is defined as above, and *O* is the observed number of significant *cis*-QTL and eGene within the same TAD. A significant chi-squared test was defined as a corresponding chi-square test P-value <0.001. There were 96 independent chi-squared tests (2 types of *cis*-QTLs × 2 cell types × 4 significant thresholds × 6 sets of finalised putative bovine TADs).

## Additional files

Additional file 1: **Table S1.** Summary of genomic conversion of mammalian TADs to the bovine genome. This table summarises results of converting mammalian TAD coordinates to the bovine genome in each step. Query: the input to liftOver in every step of genomic conversion. Target: the output in every step of genomic conversion. NA: not applicable. Gap: the genomic interval that are not marked as TAD in the data. (XLSX 495 kb)

Additional file 2: **Table S2.** Putative bovine TAD genomic coordinates. This table provides the genomic coordinates of putative bovine TADs. First and second columns (i.e. input TAD cell/tissue type and input TAD reference genome) specify the input bovine TAD set. Third, fourth and fifth columns respectively specify the chromosome number, start and end positions of the putative bovine TADs. (XLSX 22 kb)

Additional file 3: **Appendix 1.** Summary files from MEME-ChIP and FIMO. Summary file from MEME-ChIP program shows that 4 out of 82 CTCF binding motif profiles are validated in public databases of known transcription factor binding motifs, and the rest are novel CTCF binding motifs. Summary file from FIMO program shows the matched CTCF binding motifs in the bovine genome. The S2_Appendix/fimo.html and S2_Table are linked by the “motif number”, “motif width” and “best possible match” columns. (ZIP 108 kb)

Additional file 4: **Table S3.** Rank and fold change of enrichment of biological hallmarks at putative bovine TAD boundaries. This table shows the level of enrichment of each biological hallmark at putative bovine TAD boundaries. The level of enrichment for each biological signal is measured by the number of base pairs that a biological signal overlapping with the TAD boundary. Presented are two types of TAD boundaries for each TAD set, differed by whether a boundary is considered present if two neighbouring putative bovine TADs overlap each other by 1 bp. “< 0.0001” denotes the degree of overlap between the biological hallmark and TAD boundaries larger than all permuted cases. (A) The degrees of enrichment of all biological signals at the boundary of each TAD set are shown. (B) The degrees of enrichment of each pattern of CTCF binding motifs at the boundary of each TAD set are shown. Each motif pattern has a unique motif number defined by the FIMO output. Each best possible match motif is manually compared with the MEME-ChIP to assess whether the matched motif is previously reported. (XLSX 54 kb)

Additional file 5: **Table S4.** Enrichment of significant aseQTLs and eQTLs within bovine biological signals. This table shows the level of enrichment of each type of significant quantitative trait loci (QTLs) at biological hallmarks. Significant aseQTLs/eQTLs are defined by the *P*-value from aseQTL/eQTL mapping, and four P-value thresholds were tested (from **10**^***−*5**^ to **10**^***−*8**^). “< 0.0001” denotes the degree of overlap between the significant aseQTLs/eQTLs and the biological hallmark larger than all permuted cases. (XLSX 19 kb)

Additional file 6: **Table S5.** EGenes are often located within the same TAD as their furthermost and most-significant aseQTL and eQTL. This table provides the results of Chi-Square test. The Chi-Square test was to examine whether the observed frequency of eGene and its linearly-furthermost, and most-significant, aseQTL/eQTL locating in the same TAD was more often than expected. The first column specifies the putative bovine TAD set. The second column specifies the bovine cell type where the aseQTL or eQTL was measured from. A minimum *P*-value threshold was applied to require the most-significant aseQTL and eQTL passing the P-value threshold in the aseQTL/eQTL mapping, and four P-value thresholds were tested (from **10**^***−*5**^ to **10**^***−*8**^). The expected number of the significant eSNP and eGene within the same TAD was defined as the product among the maximum number of eSNPs in a chromosome analysed during aseQTL or eQTL mapping, the number of eGenes overlapping a TAD in a TAD set, and the P-value threshold that filter the significant aseQTL/eQTL mapping results. The observed number of linearly-furthermost, and most-significant, aseQTL/eQTL and eGene within the same TAD was calculated from the data. The Chi-Square test *P*-values were all much smaller than **10**^***−*8**^, and were denoted as “<10e-08” in the table. (XLSX 15 kb)

Additional file 7: **Appendix** 2. ANOVA testing the effects of TAD, CTCF and gene on ASE variation. TAD and CTCF remained significant when gene was fitted into a categorical variable in the ANOVA model, indicating that TAD and CTCF were independent factors from gene that were predictive of ASE variation. (ZIP 78 kb)

Additional file 8: **Figure S1.** Runs of genes with allele-specific expression within regulatory units. Only runs of genes within TAD and between CTCF binding motifs are shown. Our ANOVA models showed that both TAD and CTCF are significant factors (P-value ≤ **10**^***−*6**^ and false discovery rate < 0.01) explaining the observed ASE variation, while gene is a also a significant factor in Figure S1A but not a significant factor in Figure S1B. The ASE scores (y-axis) for heterozygous loci (x-axis) are plotted as black dot points in each Manhattan plot. Since ASE score was a division of paternal to maternal allelic read counts, the heterozygous locus whose ASE score is larger than 0 favours paternal expression, and the heterozygous locus whose ASE score is less than 0 favours maternal expression. The putative bovine TAD is represented as a rectangle centring at y = 0. The CTCF binding motifs (motif score ≥ 80 and motif P-value ≤ **10**^***−*8**^) are represented as the start and end position of each arrowed curve, where the direction of the arrow is the direction of transcription. Genes are represented as coloured bars starting from y = 0 towards either the top (gene on forward strand) or the bottom (gene on reverse strand) of the graph. Gene names or IDs from Ensembl UMD3.1 annotation (release 75) are listed in legend. (ZIP 260 kb)

Additional file 9: **Figure S2.** A schematic workflow of TAD mapping. In any step of the mapping and recovery procedure, a query TAD could have five possible outcomes. Only in the case of close intra-chromosomal split that TAD fragments in the target genome were merged. (PNG 203 kb)

Additional file 10: **Figure S3.** TADs, TAD boundaries and ‘knot’. (1) A graphical representation of TAD, TAD boundaries, ‘knot’ and unorganised chromatin is presented. Also presented is a graphical representation of biological hallmarks that are enriched in TAD boundaries. (2) Presented is how our graphical presentation of TAD, TAD boundaries and ‘knot’ relates to the TAD, TAD boundaries and ‘knot’ from Hi-C data. (PNG 325 kb)

## Abbreviations

ANOVA: Analysis of variance
ASE: Allele-specific expression
aseQTL: Allele-specific expression quantitative trait loci
bp: Base pair
ChIP-Seq: Chromatin immunoprecipitation (ChIP) with sequencing
CTCF: CCCTC binding factor
ECDF: Empirical cumulative distribution
ENCODE: Encyclopedia of DNA Elements
eQTL: Expression quantitative trait loci
Hi-C: High-throughput chromosome conformation capture
LD: Linkage disequilibrium
SD: Standard deviation
SINE: Short interspersed nuclear element
SNP: Single nucleotide polymorphism
TAD: Topological association domain
tRNA: Transfer RNA

## References

[1] Daetwyler HD, Capitan A, Pausch H, Stothard P, van Binsbergen R, Brondum RF (2014). Whole-genome sequencing of 234 bulls facilitates mapping of monogenic and complex traits in cattle. Nat Genet.

[2] Hayes B, Goddard M (2010). Genome-wide association and genomic selection in animal breedingThis article is one of a selection of papers from the conference “exploiting genome-wide Association in Oilseed Brassicas: a model for genetic improvement of major OECD crops for sustainable farming”. Genome.

[3] Battle A, Mostafavi S, Zhu X, Potash JB, Weissman MM, McCormick C (2014). Characterizing the genetic basis of transcriptome diversity through RNA-sequencing of 922 individuals. Genome Res.

[4] Crowley JJ, Zhabotynsky V, Sun W, Huang S, Pakatci IK, Kim Y (2015). Analyses of allele-specific gene expression in highly divergent mouse crosses identifies pervasive allelic imbalance. Nat Genet.

[5] GTEx Consortium, Lead analysts:, Laboratory, Data Analysis & Coordinating Center (LDACC):, NIH program management:, Biospecimen collection:, et al. Genetic effects on gene expression across human tissues. Nature 2017;550(7675):204–213. 10.1038/nature24277 PMC5776756 29022597

[6] Whitington T, Gao P, Song W, Ross-Adams H, Lamb AD, Yang Y (2016). Gene regulatory mechanisms underpinning prostate cancer susceptibility. Nat Genet.

[7] GTEx Consortium (2015). The genotype-tissue expression (GTEx) pilot analysis: multitissue gene regulation in humans. Science (New York, NY).

[8] Lieberman-Aiden E, van Berkum NL, Williams L, Imakaev M, Ragoczy T, Telling A (2009). Comprehensive mapping of long-range interactions reveals folding principles of the human genome. Science.

[9] Sexton T, Yaffe E, Kenigsberg E, Bantignies F, Leblanc B, Hoichman M (2012). Three-dimensional folding and functional organization principles of the Drosophila genome. Cell.

[10] Belton J-M, McCord RP, Gibcus JH, Naumova N, Zhan Y, Dekker J (2012). Hi–C: a comprehensive technique to capture the conformation of genomes. Methods.

[11] Li G, Fullwood MJ, Xu H, Mulawadi FH, Velkov S, Vega V (2010). ChIA-PET tool for comprehensive chromatin interaction analysis with paired-end tag sequencing. Genome Biol.

[12] Ong C-T, Corces VG (2014). CTCF: an architectural protein bridging genome topology and function. Nat Rev Genet.

[13] Lupiáñez DG, Kraft K, Heinrich V, Krawitz P, Brancati F, Klopocki E (2015). Disruptions of topological chromatin domains cause pathogenic rewiring of gene-enhancer interactions. Cell.

[14] Dixon JR, Selvaraj S, Yue F, Kim A, Li Y, Shen Y (2012). Topological domains in mammalian genomes identified by analysis of chromatin interactions. Nature.

[15] Rao SSP, Huntley MH, Durand NC, Stamenova EK, Bochkov ID, Robinson JT (2015). A 3D map of the human genome at Kilobase resolution reveals principles of chromatin looping. Cell.

[16] Rudan MV, Barrington C, Henderson S, Ernst C, Odom DT, Tanay A (2015). Comparative hi-C reveals that CTCF underlies evolution of chromosomal domain architecture. Cell Rep.

[17] Krijger PHL, de Laat W (2016). Regulation of disease-associated gene expression in the 3D genome. Nat Rev Mol Cell Biol.

[18] Valton A-L, Dekker J (2016). TAD disruption as oncogenic driver. Curr Opin Genet Dev.

[19] Yaffe E, Tanay A (2011). Probabilistic modeling of hi-C contact maps eliminates systematic biases to characterize global chromosomal architecture. Nat Genet.

[20] Bortle KV, Nichols MH, Li L, Ong C-T, Takenaka N, Qin ZS (2014). Insulator function and topological domain border strength scale with architectural protein occupancy. Genome Biol.

[21] Gaszner M, Felsenfeld G (2006). Insulators: exploiting transcriptional and epigenetic mechanisms. Nat Rev Genet.

[22] Mishiro T, Ishihara K, Hino S, Tsutsumi S, Aburatani H, Shirahige K (2009). Architectural roles of multiple chromatin insulators at the human apolipoprotein gene cluster. EMBO J.

[23] Ghavi-Helm Y, Klein FA, Pakozdi T, Ciglar L, Noordermeer D, Huber W (2014). Enhancer loops appear stable during development and are associated with paused polymerase. Nature.

[24] Filippova GN, Fagerlie S, Klenova EM, Myers C, Dehner Y, Goodwin G (1996). An exceptionally conserved transcriptional repressor, CTCF, employs different combinations of zinc fingers to bind diverged promoter sequences of avian and mammalian c-myc oncogenes. Mol Cell Biol.

[25] Schmidt D, Schwalie Petra C, Wilson Michael D, Ballester B, Gonçalves Â, Kutter C (2012). Waves of retrotransposon expansion remodel genome organization and CTCF binding in multiple mammalian lineages. Cell.

[26] Poulos RC, Thoms JAI, Guan YF, Unnikrishnan A, Pimanda JE, Wong JWH (2016). Functional mutations form at CTCF-Cohesin binding sites in melanoma due to uneven nucleotide excision repair across the motif. Cell Rep.

[27] Bailey TL, Johnson J, Grant CE, Noble WS (2015). The MEME suite. Nucleic Acids Res.

[28] Kim TH, Abdullaev ZK, Smith AD, Ching KA, Loukinov DI, Green Roland D (2007). Analysis of the vertebrate insulator protein CTCF-binding sites in the human genome. Cell.

[29] Maurano MT, Wang H, Kutyavin T, Stamatoyannopoulos JA (2012). Widespread site-dependent buffering of human regulatory polymorphism. PLoS Genet.

[30] Hashimoto H, Wang D, Horton JR, Zhang X, Corces VG, Cheng X (2017). Structural basis for the versatile and methylation-dependent binding of CTCF to DNA. Mol Cell.

[31] Chamberlain AJ, Vander Jagt CJ, Hayes BJ, Khansefid M, Marett LC, Millen CA (2015). Extensive variation between tissues in allele specific expression in an outbred mammal. BMC Genomics.

[32] Chamberlain AJ, Hayes BJ, Xiang R, Jagt CJV, Reich CM, MacLeod IM (2018). Identification of genetic variation regulating gene expression in dairy cattle with RNA sequence data. 11th world congress on genetics applied to livestock production.

[33] Bouwman AC, Daetwyler HD, Chamberlain AJ, Ponce CH, Sargolzaei M, Schenkel FS (2018). Meta-analysis of genome-wide association studies for cattle stature identifies common genes that regulate body size in mammals. Nature Genet.

[34] Andersson L, Archibald AL, Bottema CD, Brauning R, Burgess SC, Burt DW (2015). Coordinated international action to accelerate genome-to-phenome with FAANG, the functional annotation of animal genomes project. Genome Biol.

[35] Fullwood MJ, Liu MH, Pan YF, Liu J, Xu H, Mohamed YB (2009). An oestrogen-receptor-α-bound human chromatin interactome. Nature.

[36] Tang Z, Luo OJ, Li X, Zheng M, Zhu JJ, Szalaj P (2015). CTCF-mediated human 3D genome architecture reveals chromatin topology for transcription. Cell.

[37] Maurano MT, Haugen E, Sandstrom R, Vierstra J, Shafer A, Kaul R (2015). Large-scale identification of sequence variants influencing human transcription factor occupancy in vivo. Nat Genet.

[38] Gaffney DJ, Veyrieras J-B, Degner JF, Pique-Regi R, Pai AA, Crawford GE (2012). Dissecting the regulatory architecture of gene expression QTLs. Genome Biol.

[39] Gómez-Díaz E, Corces VG (2014). Architectural proteins: regulators of 3D genome organization in cell fate. Trends Cell Biol.

[40] Grubert F, Zaugg Judith B, Kasowski M, Ursu O, Spacek Damek V, Martin Alicia R (2015). Genetic control of chromatin states in humans involves local and distal chromosomal interactions. Cell.

[41] Speed D, Balding DJ (2014). MultiBLUP: improved SNP-based prediction for complex traits. Genome Res.

[42] MacLeod IM, Bowman PJ, Vander Jagt CJ, Haile-Mariam M, Kemper KE, Chamberlain AJ (2016). Exploiting biological priors and sequence variants enhances QTL discovery and genomic prediction of complex traits. BMC Genomics.

[43] Nguyen QH, Tellam RL, Naval-Sanchez M, Porto-Neto LR, Barendse W, Reverter A (2018). Mammalian genomic regulatory regions predicted by utilizing human genomics, transcriptomics, and epigenetics data. GigaScience.

[44] Shen Y, Yue F, McCleary DF, Ye Z, Edsall L, Kuan S (2012). A map of the cis-regulatory sequences in the mouse genome. Nature.

[45] Gentleman RC, Carey VJ, Bates DM, Bolstad B, Dettling M, Dudoit S (2004). Bioconductor: open software development for computational biology and bioinformatics. Genome Biol.

[46] Kuhn RM, Haussler D, Kent WJ (2013). The UCSC genome browser and associated tools. Brief Bioinform.

[47] Morgan M, Carlson M, Tenenbaum D, Arora S (2017). AnnotationHub: Client to access AnnotationHub resources. R package version 2.10.1.

[48] Lawrence M, Gentleman R, Carey V (2009). rtracklayer: an R package for interfacing with genome browsers. Bioinformatics.

[49] Lawrence M, Huber W, Pagès H, Aboyoun P, Carlson M, Gentleman R (2013). Software for computing and annotating genomic ranges. PLoS Comput Biol.

[50] Ma W, Noble WS, Bailey TL (2014). Motif-based analysis of large nucleotide data sets using MEME-ChIP. Nat Protoc.

[51] The UniProt Consortium (2017). UniProt: the universal protein knowledgebase. Nucleic Acids Res.

[52] Grant CE, Bailey TL, Noble WS (2011). FIMO: scanning for occurrences of a given motif. Bioinformatics.

[53] Eisenberg E, Levanon EY (2013). Human housekeeping genes, revisited. Trends Genet.

[54] Villar D, Berthelot C, Aldridge S, Rayner Tim F, Lukk M, Pignatelli M (2015). Enhancer evolution across 20 mammalian species. Cell.

[55] Zhao C, Carrillo JA, Tian F, Zan L, Updike SM, Zhao K (2015). Genome-wide H3K4me3 analysis in Angus cattle with divergent tenderness. PLoS One.

[56] Wang M, Hancock TP, MacLeod IM, Pryce JE, Cocks BG, Hayes BJ (2017). Putative enhancer sites in the bovine genome are enriched with variants affecting complex traits. Genet Sel Evol.

[57] Degner JF, Marioni JC, Pai AA, Pickrell JK, Nkadori E, Gilad Y (2009). Effect of read-mapping biases on detecting allele-specific expression from RNA-sequencing data. Bioinformatics.

