# Supplementary material for: Putative bovine topological association domains and CTCF binding motifs can reduce the search space for causative regulatory variants of complex traits
Source: BMC Genomics. 2018 May 24;19:395. doi: 10.1186/s12864-018-4800-0 (PMC5968476; doi:10.1186/s12864-018-4800-0)
Supplement: Supplementary file 3 — Appendix 1. Summary files from MEME-ChIP and FIMO. Summary file from MEME-ChIP program shows that 4 out of 82 CTCF binding motif profiles are validated in public databases of known transcription factor binding motifs, and the rest are novel CTCF binding motifs. Summary file from FIMO program shows the matched CTCF binding motifs in the bovine genome. The S2_Appendix/fimo.html and S2_Table are linked by the “motif number”, “motif width” and “best possible match” columns. (ZIP 108 kb) [file 12864_2018_4800_MOESM3_ESM.zip › S1_Appendix/fimo.html]

FIMO Results


---

|  |  |  |
| --- | --- | --- |
| **Database and Motifs** | **High-scoring Motif Occurences** | **Debugging Information** |

  
  


---
