# Supplementary material for: Putative bovine topological association domains and CTCF binding motifs can reduce the search space for causative regulatory variants of complex traits
Source: BMC Genomics. 2018 May 24;19:395. doi: 10.1186/s12864-018-4800-0 (PMC5968476; doi:10.1186/s12864-018-4800-0)
Supplement: Supplementary file 7 — Appendix 2. ANOVA testing the effects of TAD, CTCF and gene on ASE variation. TAD and CTCF remained significant when gene was fitted into a categorical variable in the ANOVA model, indicating that TAD and CTCF were independent factors from gene that were predictive of ASE variation. (ZIP 78 kb) [file 12864_2018_4800_MOESM7_ESM.zip › S2_Appendix/S2_Appendix.docx]

# Results

## ANOVA testing the effects of TAD, CTCF and gene on ASE variation

Analysis of variance (ANOVA) was performed to test the effects of TAD, CTCF and gene on the variation of allele-specific expression (ASE) among regulatory units. Chamberlain *et al.* [1] observed runs of genes were expressed in favour of the same parental chromosome, so we expected and observed significant gene effects from the ANOVA model (Si Table). TAD was a significant factor (P-value ≤ ${10}^{-8}$) in both models among all 108 cohorts tested. CTCF was a significant factor (P-value ≤ ${10}^{-8}$) in both ANOVA models in 77 cohorts. Gene was a significant factor (P-value ≤ ${10}^{-8}$) in 102 cohorts in Model (B). There were 74 cohorts, where all factors fitted were all significant in both models (Si Table). This indicated that TAD and CTCF contributed to ASE variation independent of gene effects.

# Methods

## ANOVA testing the effects of TAD, CTCF and gene on ASE variation

Two analysis of variance (ANOVA) models was performed to examine the effects of TAD, CTCF and gene on the variation of allele-specific expression (ASE) among regulatory units. ASE scores, which was defined as a division of paternal to maternal allelic read counts in a tissue (same definition as that in manuscript), were still fitted as the response in both models, but the categorical variables fitted to each model were different:

- Model (A) was a TAD + CTCF model, where TAD and CTCF gap were both fitted as categorical variables in the ANOVA model. CTCF gaps were required to be embedded within the putative bovine TADs. This is same model as Model (3) in the manuscript.
- Model (B) was a TAD + CTCF + Gene model, where TAD, CTCF gap and gene were fitted as the categorical variables in the ANOVA model. CTCF gaps were embedded within the putative bovine TADs. Genes overlapped with TAD and/or CTCF gap.

In each ANOVA model, all innermost genomic regions were required to contain at least 5 heterozygous exonic SNPs that all had valid ASE scores. There were 108 independent tests performed from each ANOVA model, which were from the combination of 6 sets of finalised putative bovine TADs and 18 bovine tissues. The significance of an ANOVA test was declared at P-value ≤ ${10}^{-8}$.

The permutation tests, with 10,000 repeats, were performed to test whether the observed ANOVA result was random. In the permutation test, the ASE scores were shuffled across the whole genome and then the model was refitted with the permuted dataset. The R-squared value from the original dataset, $R$, was compared with the 10,000 null R-squared values from the random shuffles, $R'$. A significant ANOVA cohort was declared if $R$ was larger than all $R'$ values. In a significant ANOVA test, a regulatory unit was declared to display significant ASE effects if the absolute value of the averaged ASE scores in the region, $\left| n \right|$, was larger than 99% of the absolute value of the averaged ASE scores in the permutations $|m|$, i.e. false discovery rate (FDR) < 0.01, and the p-value for $n$ was no larger than ${10}^{-6}$. There were 108 independent permutation tests performed.

# Supporting Information

## Si Table

**Compare ANOVA outputs between whether gene is fitted as a categorical variable.** The first column is the input TAD set. The second column is the bovine tissue where allele-specific expression was measured from. The rest of the columns are ANOVA outputs, including the categorical variable in the ANOVA model, the degrees of freedom, the sum of squares, the mean of squares, the F-statistics and the P-value. ANOVA results from the model where only TAD and CTCF gap were fitted, are listed under columns with header as “TAD + CTCF”. ANOVA results from the model where TAD, CTCF gap and gene were all fitted, are listed under columns with header as “TAD + CTCF + Gene”. “NA” denotes the cases where no results should apply.

# Reference

1. Chamberlain AJ, Vander Jagt CJ, Hayes BJ, Khansefid M, Marett LC, Millen CA, et al. Extensive variation between tissues in allele specific expression in an outbred mammal. BMC Genomics. 2015;16(1):993.
