# Supplementary figures and images for: Putative bovine topological association domains and CTCF binding motifs can reduce the search space for causative regulatory variants of complex traits
Source: BMC Genomics. 2018 May 24;19:395. doi: 10.1186/s12864-018-4800-0 (PMC5968476; doi:10.1186/s12864-018-4800-0)

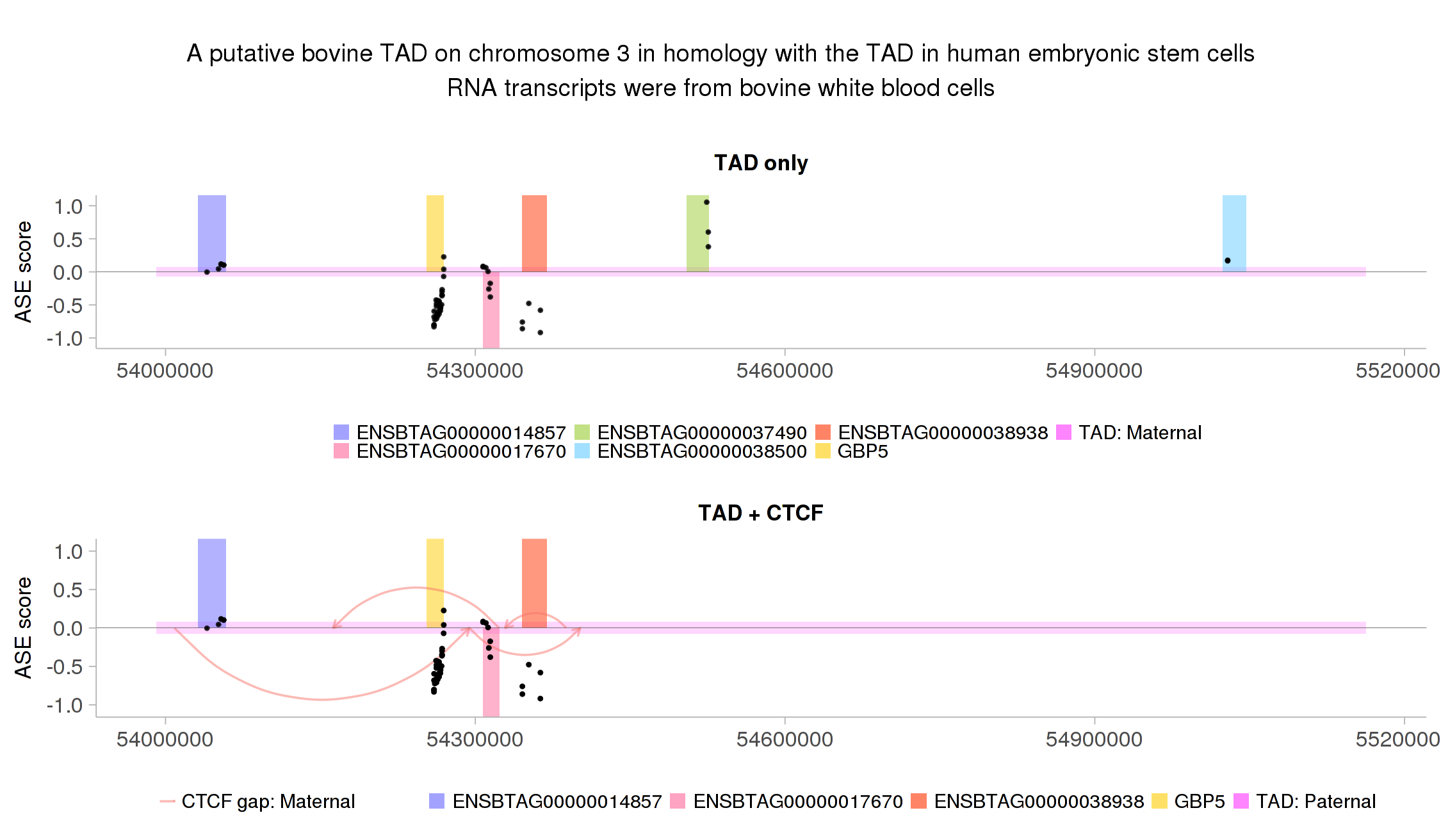

Supplement: Supplementary file 8 — Figure S1. Runs of genes with allele-specific expression within regulatory units. Only runs of genes within TAD and between CTCF binding motifs are shown. Our ANOVA models showed that both TAD and CTCF are significant factors (P-value ≤ 10−6 and false discovery rate < 0.01) explaining the observed ASE variation, while gene is a also a significant factor in Figure S1A but not a significant factor in Figure S1B. The ASE scores (y-axis) for heterozygous loci (x-axis) are plotted as black dot points in each Manhattan plot. Since ASE score was a division of paternal to maternal allelic read counts, the heterozygous locus whose ASE score is larger than 0 favours paternal expression, and the heterozygous locus whose ASE score is less than 0 favours maternal expression. The putative bovine TAD is represented as a rectangle centring at y = 0. The CTCF binding motifs (motif score ≥ 80 and motif P-value ≤ 10−8) are represented as the start and end position of each arrowed curve, where the direction of the arrow is the direction of transcription. Genes are represented as coloured bars starting from y = 0 towards either the top (gene on forward strand) or the bottom (gene on reverse strand) of the graph. Gene names or IDs from Ensembl UMD3.1 annotation (release 75) are listed in legend. (ZIP 260 kb) [file 12864_2018_4800_MOESM8_ESM.zip › S1A_Figure.png]

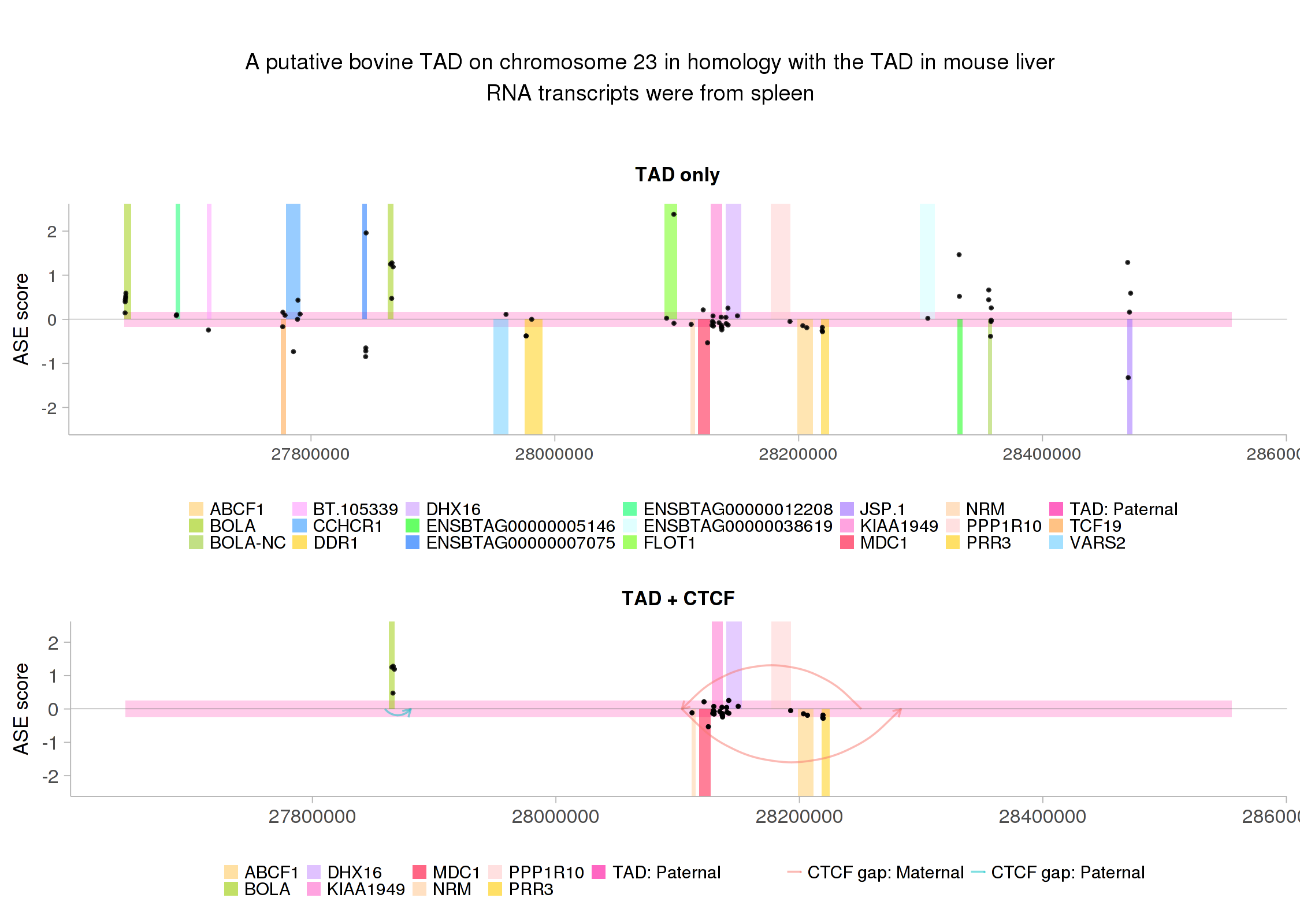

Supplement: Supplementary file 8 — Figure S1. Runs of genes with allele-specific expression within regulatory units. Only runs of genes within TAD and between CTCF binding motifs are shown. Our ANOVA models showed that both TAD and CTCF are significant factors (P-value ≤ 10−6 and false discovery rate < 0.01) explaining the observed ASE variation, while gene is a also a significant factor in Figure S1A but not a significant factor in Figure S1B. The ASE scores (y-axis) for heterozygous loci (x-axis) are plotted as black dot points in each Manhattan plot. Since ASE score was a division of paternal to maternal allelic read counts, the heterozygous locus whose ASE score is larger than 0 favours paternal expression, and the heterozygous locus whose ASE score is less than 0 favours maternal expression. The putative bovine TAD is represented as a rectangle centring at y = 0. The CTCF binding motifs (motif score ≥ 80 and motif P-value ≤ 10−8) are represented as the start and end position of each arrowed curve, where the direction of the arrow is the direction of transcription. Genes are represented as coloured bars starting from y = 0 towards either the top (gene on forward strand) or the bottom (gene on reverse strand) of the graph. Gene names or IDs from Ensembl UMD3.1 annotation (release 75) are listed in legend. (ZIP 260 kb) [file 12864_2018_4800_MOESM8_ESM.zip › S1B_Figure.png]

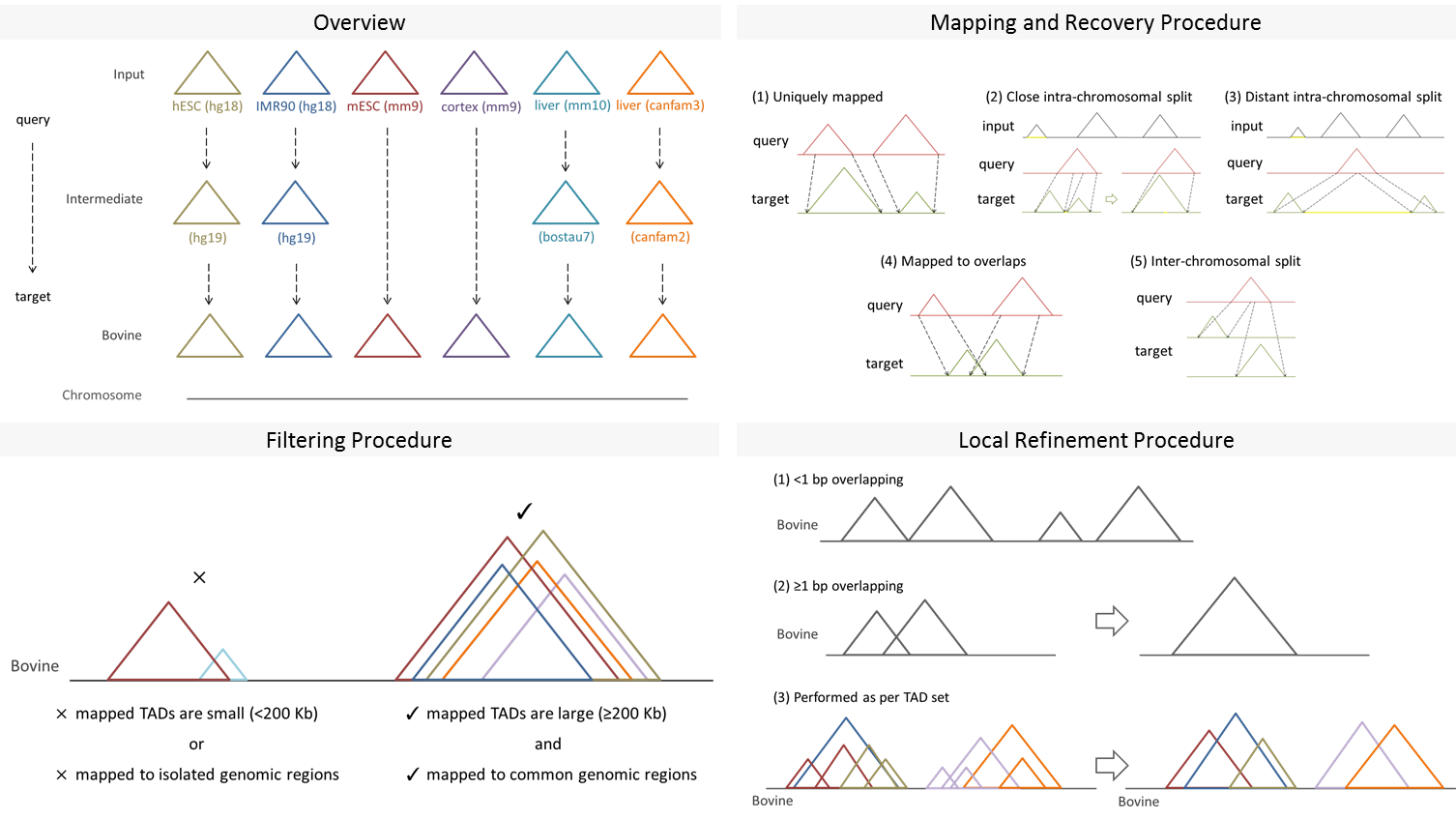

Supplement: Supplementary file 9 — Figure S2. A schematic workflow of TAD mapping. In any step of the mapping and recovery procedure, a query TAD could have five possible outcomes. Only in the case of close intra-chromosomal split that TAD fragments in the target genome were merged. (PNG 203 kb) [file 12864_2018_4800_MOESM9_ESM.png]

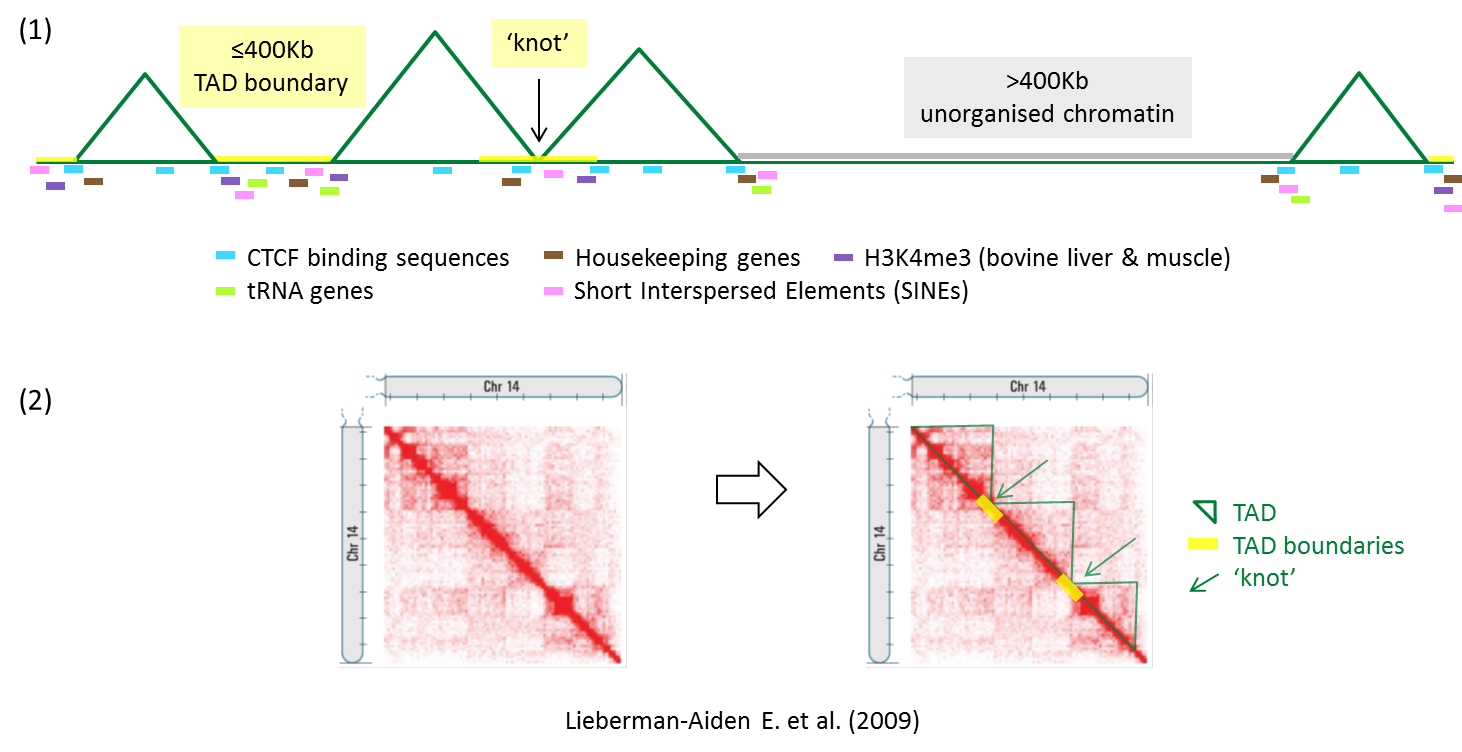

Supplement: Supplementary file 10 — Figure S3. TADs, TAD boundaries and ‘knot’. (1) A graphical representation of TAD, TAD boundaries, ‘knot’ and unorganised chromatin is presented. Also presented is a graphical representation of biological hallmarks that are enriched in TAD boundaries. (2) Presented is how our graphical presentation of TAD, TAD boundaries and ‘knot’ relates to the TAD, TAD boundaries and ‘knot’ from Hi-C data. (PNG 325 kb) [file 12864_2018_4800_MOESM10_ESM.png]
